# Preparation and Electrochromic Properties of Benzodithiophene-Isoindigo Conjugated Polymers with Oligoethylene Glycol Side Chains

**DOI:** 10.3390/ma16010060

**Published:** 2022-12-21

**Authors:** Qilin Wang, Yuehui Zhai, Danming Chao, Zheng Chen, Zhenhua Jiang

**Affiliations:** 1 Engineering Research Center of Special Engineering Plastics, Ministry of Education, National and Local Joint Engineering Laboratory for Synthetic Technology of High Performance Polymer, College of Chemistry, Jilin University, Changchun 130012, China; 2 School of Materials and Energy, University of Electronic Science and Technology of China, Chengdu 611731, China

**Keywords:** electrochromism, oligoethylene glycol (OEG), green solvent, benzodithiophene-isoindigo

## Abstract

Functional polymers featuring good processability in non-halogenated, benzene-free green solvents are highly desired due to health and environmental concerns. Herein, a series of novel D-A type conjugated polymers, PBDT-IIDs, are designed and successfully prepared by “green” functionalization of the polymers with highly hydrophilic, highly polar, highly flexible, and biocompatible oligoethylene glycol (OEG) side chains in order to improve the processability. These series polymers are named PBDT-IID2, PBDT-IID3, and PBDT-IID4, respectively, according to the number of oxygen atoms in the side chain. After confirmation by structural characterization, the basic properties of PBDT-IIDs are also investigated. With the increase in the OEG side chain length, the polymer PBDT-IID4 not only has good solubility in the halogen solvent chlorobenzene, but also exhibits excellent solubility in the green halogen-free solvent methyltetrahydrofuran (Me-THF). As a result, the green solvent Me-THF can also be applied to prepare PBDT-IIDs’ electrochromic active layers, except for chlorobenzene and toluene. The electrochromism of PBDT IIDs under both positive and negative voltages has a practical application potential. The several controllable switches between dark green and khaki (0–0.6 V) are expected to show great potential in the field of military camouflage. Furthermore, according to the principle of red, green, and blue (RGB) mixing, light blue-green in the reduced state (−1.6 V) can be used in the preparation of complementary ECDs to provide one of the three primary colors (green).

## 1. Introduction

Electrochromic materials that can reversibly change color under an applied voltage are considered one of the most promising smart materials in the fields of bionic technology, infrared stealth, military camouflage, energy storage, and flexible wearable devices [1,2,3,4,5,6,7,8]. Among many candidate EC materials, electrochromic polymers (ECPs) are preferred for many of these applications due to their fast-switching speed, abundant color change, low processing cost, excellent mechanical properties, and easily adjustable performance through structural design [9,10,11]. Although the widely studied donor-acceptor type electrochromic conjugated polymers have a low starting potential, the highly conjugated backbone structure often leads to a mostly unsatisfactory solubility for simple solvent processing methods (spin coating, scraping, spraying, etc.) [12,13]. In this context, side chain engineering studies on conjugated polymers with the aim of increasing solubility are an effective way to solve this problem. To address the above challenges, scientists have proposed a feasible solution strategy to increase the solubility of polymers in greener polar or non-halogenated non-aromatic solvents by introducing large polar groups or chain segment structures into the side-chains of D-A conjugated polymers, thus achieving the ideal industrial production method of polymer processing and preparation with green solvents such as water and alcohols [14,15,16,17].

Among the many high-performance conjugated polymer materials known so far, the donor-acceptor combination with isoindigo (IID) as the acceptor unit and benzodithiophene (BDT) as the donor unit has impressed researchers due to its excellent optoelectronic properties [18,19,20]. Isoindigo is a natural pigment raw material whose molecular structure is composed of two 2-hydroxyindoles containing electron-deficient lactam rings linked by double bonds; the whole molecular unit not only has a large planar conjugated structure but also has an extremely strong electron-pulling ability. So, isoindigo is an ideal conjugated thick ring structure acceptor unit [19]. Benzodithiophene, a “star” structural unit in the field of organic optoelectronic materials, is a conjugated polycyclic molecular unit consisting of two thiophene rings and a central benzene ring that is often used as a donor structural unit in the preparation and study of high-performance D-A conjugated polymers [20]. All of the literature work reported so far has been done to increase the solubility of PBDT-IID by linking linear/branched low-polarity alkane/aromatic/heterocyclic side chains to the main chain of the PBDT-IID. PBDT-IIDs based on non-polar alkane side chain modification were fully soluble in halogenated solvents or non-polar non-halogenated aromatic solvents and were successfully used for subsequent processing and production, but their solubility in polar non-halogenated solvents still needs to be improved because the batch use of the above solvents undoubtedly poses a great threat to the ecological environment and human health.

Oligomeric ethylene glycol side chains are highly hydrophilic, hyperpolar, flexible, and biocompatible compared to low-polar side chains. Furthermore, D-A conjugated polymers containing OEG side chains have better intermolecular chain stacking than D-A conjugated polymers containing low-polarity side chains in the thin film state, which facilitates the carrier transfer between polymer molecular chains. A series of D-A conjugated polymers containing OEG side chains have been developed and employed in the field of organic electronics. For example, Prof. Deqing Zhang’s group synthesized a diketopyrrole-thiophene conjugated polymer containing OEG side chains, and the experimental data showed that the hole mobility of the diketopyrrole-thiophene conjugated polymer containing OEG side chains was much higher than that of the diketopyrrole-thiophene conjugated polymer containing alkyl side chains [21]. The OEG side chains were incorporated into the conjugated backbone of PIBET, and the related organic electronic device OECT was prepared by the team led by Prof. Yue L. [22]. It was found that the PIBET modified with the OEG side chains had a shorter response time in an aqueous electrolyte than the PIBET modified with alkyl branched chains. Moreover, the PIBET with OEG side chains showed better adhesion to substrates (gold IDME and ITO with PET coatings) than the PIBET with alkyl-branched chains. It is also worth noting that the length, pattern, and substitution position of the side chains in the main chain of the conjugated polymer all have some influence on the photovoltaic properties of conjugated polymers containing the same structure [23].

Accordingly, three PBDT-IIDs (PBDT-IID2, PBDT-IID3, and PBDT-IID4) containing different lengths of OEG side chains were designed and synthesized by the “green” functionalization of PBDT-IID polymers with oligoethylene glycol chain segments to achieve PBDT-IIDs polymers that are soluble in non-halogenated benzene-free green solvents (limonene, cyclopentane methyl ether, methyl tetrahydrofuran, etc.). That is, while retaining the dissolution and processing performance of the material, it reduces the harm of its production process to the environment and human body. In the subsequent performance studies, it was found that the thermal stability and solubility of the three PBDT-IID polymers were excellent, as evidenced by the T_d5%_ above 300 °C and the ability to dissolve them in weakly polar halogen-free solvents, both of which increased with the length of the OEG side chain. Furthermore, all the three polymers have the ability to undergo electrochromic color change under both positive and negative voltage scans. In the positive voltage (0–0.6 V) scan, the polymer film color switches reversibly between dark green and khaki, while in the negative voltage (−1.6–0 V) scan, the film changes from dark green in the neutral state to light blue-green in the reduced state in a very short period of time. The above special color switching states make D-A conjugated PBDT-IID polymers promising for applications such as dynamic military camouflage and color-complementary mixed-color ECDs [24].

## 2. Materials and Methods

### 2.1. Materials

(E)-6,6′-Dibromo-[3,3′-diindolylidene]-2,2′-dione (97%), benzo [1,2-b:4,5-b′]dithiophene-4,8-dione (98%), trimethyltin chloride (99%, 1.0 mol/L in hexane), n-butyllithium (97%, 2.5 mol/L in hexane), sodium hydroxide (97%), zinc powder (98%, 200 mesh), diethylene glycol monomethyl ether (98%), triethylene glycol monomethyl ether (99%), tetraethyleneglycol monomethyl ether (98%), 2-hexyl-1-decanol (94%), methyl sulfonyl chloride (99%), tris(dibenzylideneacetone)dipalladium chloroform adduct (99%), tris(o-methylphenyl)phosphine (99%), tetrahydrofuran (99%, extra dry, with molecular sieves, water ≤ 50 ppm), *o*-xylene (99%, extra dry, with molecular sieves, water ≤ 50 ppm), tetrabutylammonium hexafluorophosphonate (99%), acetonitrile (99%, extra dry, with molecular sieves, water ≤ 50 ppm), and limonene (99%) were purchased from Energy Chemical Company (Anqing, China) and were not treated in any way prior to use. Other solvents, such as 2-methyltetrahydrofuran, ethyl acetate, and trichloromethane are analytically pure and purchased from Sinopharm (Shanghai, China).

### 2.2. Characterization Methods

The water contact angle was estimated using a contact angle measuring instrument, DAS25 (KRUSS, Hamburg, Germany). NMR hydrogen spectra were measured by the Bruker Avance 300 (Bruker BioSpin, Rheinstetten, Germany). The molecular weight of the polymers was determined by high temperature gel chromatography PL-GPC220 (Agilent, Palo Alto, CA, USA). The thermodynamic properties were estimated by a Q2000 thermogravimetric analyzer (Mettler, Zurich, Switzerland) and a Mettler Toledo differential scanning calorimeter (Agilent, Palo Alto, CA, USA). The spectroscopy-related tests of the materials were performed by the UV-Vis spectrometer N4/N4S (Jingke, Quzhou, China). The electrochemical performance tests were conducted on an electrochemical workstation, CHI660E (Chenhua, Shanghai, China). The tests of the electrochromic properties required the combination of the UV-Vis spectrometer and electrochemical workstation.

## 3. Design and Synthesis

### 3.1. Design

**Figure 1:**
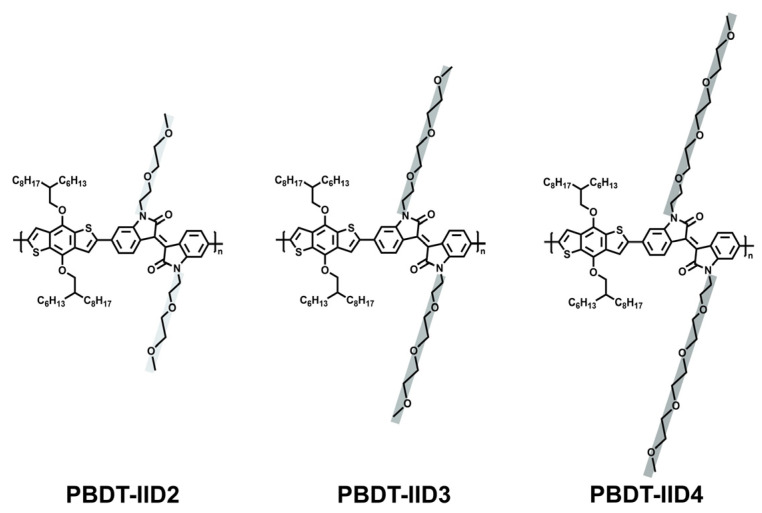
The chemical structure diagrams of PBDT-IID2, PBDT-IID3, and PBDT-IID4.

**Scheme 1:**
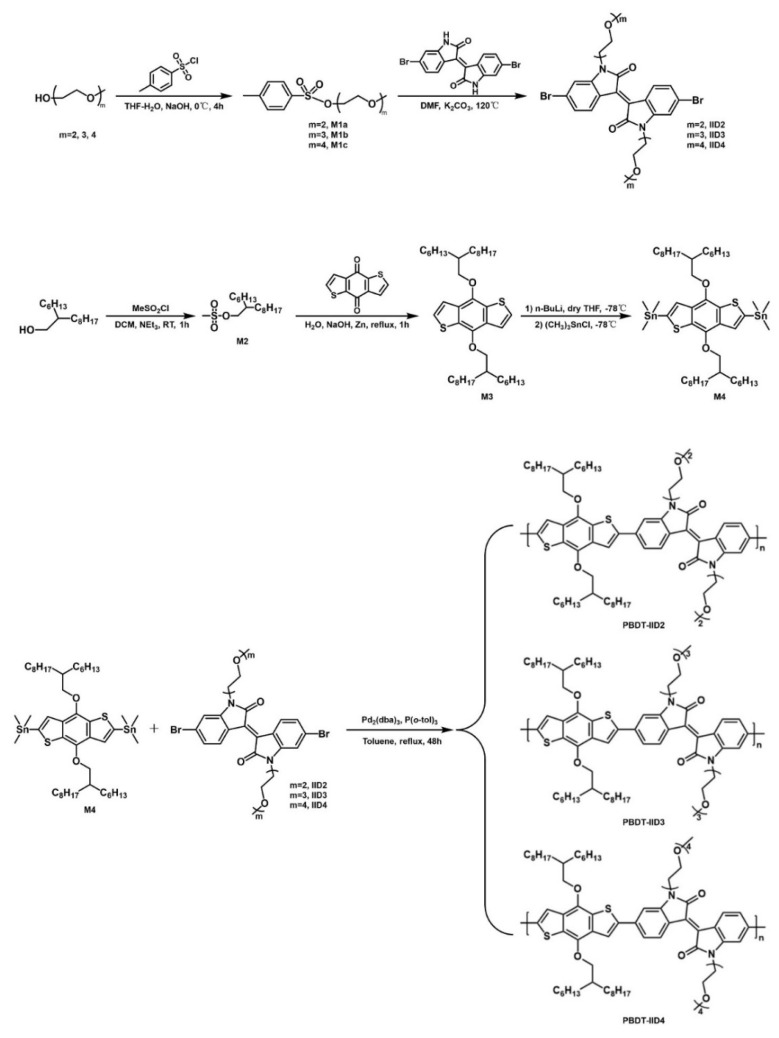
The synthesis route of monomers (M1a, M1b, M1c, M2, M3, M4, IID2, IID3 and IID4) and polymers (PBDT-IID2, PBDT-IID3 and PBDT-IID4).

Benzodithiophene/isoindigo (BDT/IID), with its perfect planar π-conjugated structure and electron rich/deficient nature, is widely used to construct narrow-band-gap semiconducting polymers such as PBDT-IID. However, most of them need to use halogenated solvents or non-polar halogen-free aromatic hydrocarbon solvents in actual production. The large-scale use of the above processing solvents inevitably brings great harm to the ecological environment and human health. Herein, to further improve the solubility of PBDT-IIDs in non-halogenated benzene-free green solvents and provide an insight on the effect of oligomeric ethylene glycol (OEG) and alkyl chains on of the side chains on PBDT-IID physical and optoelectronic properties, three derivatives of PBDT-IID (Figure 1) are synthesized via Stille polymerization of branched alkyl functionalized trimethylstannyl-bis-BDT monomer and EG functionalized dibromo-IID monomers. The synthesis routes of the three PBDT-IID polymers (PBDT-IIDs) are shown in Scheme 1, named PBDT-IID1, PBDT-IID2, and PBDT-IID3, respectively, according to the length of the OEG side chain.

### 3.2. Synthesis

#### 3.2.1. The Synthesis of Monomers M1a, M1b, and M1c

There were 10 g (83.2 mmol) of diethylene glycol monomethyl ether, 5 g (125 mmol) sodium hydroxide, 50 mL of deionized water, and 40 mL of tetrahydrofuran that were stirred at 0 °C in a 250 mL round-bottom flask under an argon atmosphere for 2 h, and then 15.8 g (83.2 mmol) of 4-toluenesulfonyl chloride was dissolved in 40 mL of tetrahydrofuran and dropped slowly into the reaction solution by a constant pressure drip funnel. After stirring for another two hours, the reaction solution was poured into 100 mL of dichloromethane, and the organic phase was repeatedly washed with brine. The organic phase was then dried overnight with anhydrous Na_2_SO_4_. Finally, the dichloromethane was removed by a rotary evaporation device, and the remaining products were purified by silicone column chromatography to obtain 17.3 g of colorless oily liquid M1a (yield: 76%). ^1^H NMR (300 MHz, CDCl_3_, δ, ppm): 7.75 (d, *J* = 8.3 Hz, 2H), 7.30 (d, *J* = 7.9 Hz, 2H), 4.12 (m, 2H), 3.64 (m, 2H), 3.53 (m, 2H), 3.43 (m, 2H), 3.30 (s, 3H), and 2.409 (s, 3H).

The synthesis step of M1b is the same as M1a, so it will not be described in detail here. ^1^H NMR (300 MHz, CDCl_3_, δ, ppm): 7.80 (d, *J* = 8.2 Hz, 2H), 7.34 (d, *J* = 7.9 Hz, 2H), 4.16 (m, 2H), 3.69 (m, 2H), 3.59 (m, 6H), 3.53 (m, 2H), 3.37 (s, 3H), and 2.44 (s, 3H).

The synthesis step of M1c is the same as M1a, therefore it will not be elaborated here. ^1^H NMR (300 MHz, CDCl_3_, δ, ppm): 7.79 (d, *J* = 8.3 Hz, 2H), 7.34 (d, *J* = 7.9 Hz, 2H), 4.15 (m, 2H), 3.65 (m, 2H), 3.60 (m, 10H), 3.52 (m, 2H), 3.36 (s, 3H), and 2.44 (s, 3H).

#### 3.2.2. The Synthesis of Monomer M2

Monomer M2 is synthesized according to the literature [22]. Under an argon atmosphere, 80 mL of anhydrous dichloromethane and 6.06 g (25 mmol) of 2-hexyl-1-decanol were added to a 250 mL round-bottom flask, and then 2.15 mL (27 mmol) of methanesulfonyl chloride was dissolved in 3.8 mL (27 mmol) of triethylamine and added to the reaction solution by dropping it through a constant pressure droplet funnel. The reaction solution was stirred at room temperature for one hour. The oily product was obtained after removing the reaction solvent by using a rotary evaporation device. Subsequently, the oily product was dissolved in 150 mL of ether, and the organic phase was washed three times with 100 mL of deionized water. After that, the organic phase was collected and dried overnight with anhydrous Na_2_SO_4_. Finally, the ether was removed by a rotary evaporation device, and the remaining products were purified by silica gel column chromatography to obtain 3.7 g of colorless oily liquid M2 (yield: 70%). ^1^H NMR (300 MHz, CDCl_3_, δ, ppm): 4.09 (d, *J* = 5.5 Hz, 2H), 2.97 (s, 3H), 1.69–1.67 (m, 1H), 1.43–1.24 (m, 22H), and 0.86 (m, 6H).

#### 3.2.3. The Synthesis of Monomer M3

There were 0.5 g (2.27 mmol) of Benzo [1,2-b:4,5-b′] dithiophene-4,8-dione, 0.386 g (5.9 mmol) of zinc powder, 0.22 g (0.681 mmol) of tetrabutylammonium bromide, 1.362 g (34 mmol) of potassium hydroxide, and 50 mL of deionized water that were added to a 50 mL round bottom flask and stirred for an hour under an argon atmosphere for refluxing. Subsequently, 2.183 g (6.81 mmol) of M2 was added to the reaction system and stirred for 2 h, then 0.148 g (2.27 mmol) of zinc powder was added again and reflux was continued for 12 h. After 12 h, the solution was poured into 30 mL of deionized water and washed with ether three times to collect the organic phase. The organic phase was then dried with anhydrous Na_2_SO_4_ overnight. Finally, the ether was removed by a rotary evaporation device and the remaining products were purified by silica gel column chromatography to obtain 1.28 g of tan oily liquid M3 (yield: 84%). ^1^H NMR (300 MHz, CDCl_3_, δ, ppm): 7.48 (d, *J* = 4.8 Hz, 2H), 7.37 (d, *J* = 4.8 Hz, 2H), 4.18 (d, *J* = 4.8 Hz, 4H), 1.86 (m, 2H), 1.33 (m, 4H), 1.69–1.67 (m, 8H), 1.32–1.29 (m, 40H), and 0.90 (m, 12H).

#### 3.2.4. The Synthesis of Monomer M4

Under an argon atmosphere, 15 mL of anhydrous tetrahydrofuran and 3.6 g (5.4 mmol) of M3 were added to a 50 mL cleanly dried round-bottom flask and stirred for 20 min at −78 °C, then the n-hexane solution of 5.2 mL (12.96 mmol) of n-butyl lithium (n-butyllithium concentration 2.5 mol/L) was slowly dropped to the reaction solution by a syringe with a 15-min stirring. The reaction was slowly warmed to room temperature, stirred at room temperature for 90 min, and again cooled to −78 °C for 15 min. The 12.96 mL (12.96 mmol) of trimethyltin chloride n-hexane solution (trimethyltin chloride concentration was 1 mol/L) was slowly added to the reaction solution by syringe and stirred for 15 min. The reaction was slowly warmed again to room temperature and stirred overnight at room temperature. At the end of the reaction, the solution was poured into 200 mL of saturated aqueous sodium fluoride solution, and the aqueous phase was extracted with dichloromethane. The organic phase was washed with deionized water after it was collected and dried overnight with anhydrous Na_2_SO_4_. Finally, dichloromethane was removed using a rotary evaporation device, and the remaining product was vacuum dried at 80 °C to give 4.9 g of light yellow solid M4 (yield: 81%). ^1^H NMR (300 MHz, CDCl_3_, δ, ppm): 7.51 (s, 2H), 4.18 (d, *J* = 4.8 Hz, 4H), 1.88 (m, 2H), 1.64 (m, 4H), 1.54–1.41 (m, 8H), 1.31–1.28 (m, 40H), 0.90 (m, 12H), and 0.44 (s, 18H).

#### 3.2.5. The Synthesis of Monomers IID2, IID3, and IID4

Under an argon atmosphere, 1.21 g (2.42 mmol) of M4, anhydrous potassium carbonate, and 40 mL N, N- dimethylformamide were added to a 100 mL round-bottom flask and stirred at room temperature for 10 min, then 1.45 g (5.324 mmol) of M1a was added to the reaction solvent and stirred at 120 °C overnight. The next day, the reacted solution was poured into a saturated saline, left to stand, and then filtered to collect the filter residue. After washing the filter residue three times, it was recrystallized in ethanol, and the solid was collected and dried overnight at 60 °C to obtain 1.15 g of red flocculent solid IID2 (yield: 75%). ^1^H NMR (300 MHz, CDCl_3_, δ, ppm): 9.02 (d, *J* = 0.6 Hz, 2H), 7.17 (m, 4H), 3.97 (m, 4H), 3.74 (m, 4H), 3.60 (m, 4H), 3.49 (m, 4H), and 3.35 (s, 6H).

The synthesis step of IID3 is the same as IID2, so it will not be described in detail here. ^1^H NMR (300 MHz, CDCl_3_, δ, ppm): 9.04 (d, *J* = 9.6 Hz, 2H), 7.16 (m, 4H), 3.97 (m, 4H), 3.74 (m, 4H), 3.60 (m, 12H), 3.49 (m, 4H), and 3.34 (s, 6H).

The synthesis step of IID4 is the same as IID2, so it will not be described in detail here. ^1^H NMR (300 MHz, CDCl_3_, δ, ppm): 9.04 (d, *J* = 9.0 Hz, 2H), 7.16 (m, 4H), 3.96 (m, 4H), 3.74 (m, 4H), 3.62 (m, 20H), 3.52 (m, 4H), and 3.36 (s, 6H).

#### 3.2.6. The Synthesis of Polymers PBDT-IID2, PBDT-IID3, and PBDT-IID4

There were 50.5 mg (0.05 mmol) of M4, 1.5 mg (0.0015 mmol) of tris(dibenzylacetone) dipalladium chloroform admixture, 1.37 mg (0.0045 mmol) of tris(o-methylphenyl) phosphine, and 31.2 mg (0.05 mmol) of IID2 that were loaded into a cleanly dried 25 mL round-bottom flask, the inside of the flask was anaerobically treated after sealing, and 5 mL of anhydrous *o*-xylene was added to the flask through a syringe. The reaction solution was stirred at 130 °C for two days, and then the reaction was stopped. Later, the reaction solution was poured into 50 mL of methanol, set aside, and filtered to collect the solid fraction. Subsequently, the solid was extracted with a Sohren’s extractor (the extracting solvents were petroleum ether, ethanol, acetone, ethyl acetate, tetrahydrofuran, and dichloromethane, in that order). Finally, solvents with high molecular weight components were removed by vacuum drying.

The yield of PBDT-IID2 is 60%. ^1^H NMR (300 MHz, CDCl_3_, δ, ppm): 9.3–8.4 (br, 2H), 7.2–6.0 (br, 6H), 3.97–3.30 (br, 22H), 1.88–0.90 (br, 66H), *M*_n_ = 54 kDa, and PDI = 2.20.

The synthesis step of PBDT-IID3 is the same as PBDT-IID2, so it will not be described in detail here. The yield of PBDT-IID3 is 52%. ^1^H NMR (300 MHz, CDCl_3_, δ, ppm): 9.3–8.4 (br, 2H), 7.2–6.0 (br, 6H), 3.97–3.30 (br, 34H), 1.88–0.90 (br, 66H), *M*_n_ = 49 kDa, and PDI = 3.67.

The synthesis step of PBDT-IID4 is the same as PBDT-IID2, so it will not be elaborated here. The yield of PBDT-IID4 is 52%. ^1^H NMR (300 MHz, CDCl_3_, δ, ppm): 9.3–8.4 (br, 2H), 7.2–6.0 (br, 6H), 3.97–3.30 (br, 42H), 1.88–0.90 (br, 66H), *M*_n_ = 37 kDa, and PDI = 4.91. The NMR spectra of the above monomers and polymers are shown in Figure S1.

## 4. Results and Discussion

### 4.1. Basic Characterization

**Figure 2:**
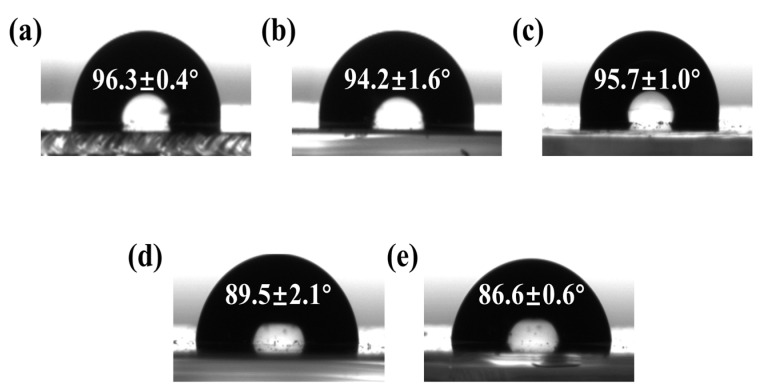
The water contact angle image: (**a**–**c**) The PBDT-IID2, PBDT-IID3, and PBDT-IID4 film formed with chlorobenzene, respectively; (**d**) The PBDT-IID4 film formed with methyl tetrahydrofuran; (**e**) The PBDT-IID4 film formed with toluene.

In order to be practically applicable, semiconducting polymers should exhibit good solubility. Table S1 lists the results of qualitative solubility testing, which was performed using 10 mg of polymer and 1 mL of solvent. These data indicate that all of the PBDT-IIDs exhibit good solubility in common chlorinated solvents such as chlorobenzene, dichloromethane, and chloroform. Due to the significant variation in the OEG side chain lengths of the three PBDT-IIDs, these polymers display markedly different solubilities in some solvents. For instance, PBDT-IID4 exhibits good solubility in the nonpolar green solvent limonene and the polar DMF, while PBDT-IID2 and PBDT-IID3 exhibit low solubility in them. This phenomenon can be explained by the improved solubility of PBDT-IIDs in halogen-free solvents via increasing the length of the OEG side chain. In addition to common chlorinated solvents, PBDT-IIDs also exhibit good solubility in some non-halogen weakly polar organic solvents (toluene) and non-halogen polar solvents (2Me-THF), which means that PBDT-IIDs show great potential for green solvent-based manufacturing of large-area, thin-film organic semiconductor devices. The water contact angle test revealed that the hydrophobicity of the three polymers was essentially the same when processed into films by the same solvent, while the hydrophilicity of the same polymer processed into films by different solvents produced significant differences. In other words, the hydrophobicity of the films was mainly determined by the type of processing solvent. Specifically, the greater the polarity of the polymer processing solvent, the more hydrophobic the film formed (Figure 2). The molecular weights (*M*_n_) for all of the PBDT-IIDs were determined by gel permeation chromatography (GPC) using dichloromethane as the eluent and polystyrene as the standard reference. The measured relative polymer molecular weights are in the range between 37 kDa and 54 kDa (Table 1). The high molecular weight not only ensures good film-forming properties of the polymer during solvent processing, but also meets the basic requirements for the molecular weight of optoelectronic materials (degree of polymerization DP ≥ 10).

### 4.2. The Thermostability Properties of PBDT-IID Polymers

**Figure 3:**
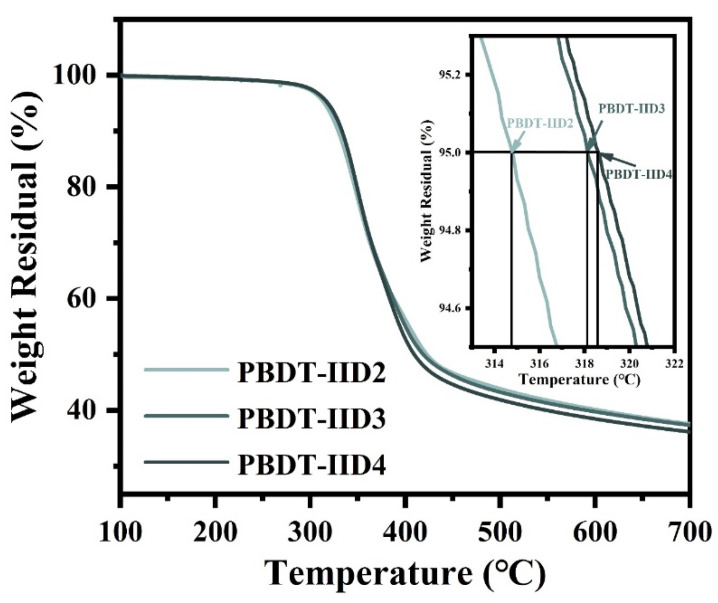
The thermogravimetric curves of PBDT-IID2, PBDT-IID3, and PBDT-IID4 under a nitrogen atmosphere.

Considering that the thermal properties of photopolymer materials are one of the main factors affecting the service life of their corresponding devices, the thermal properties of PBDT-IID2, PBDT-IID3, and PBDT-IID4 were studied by thermogravimetric analysis (TGA) and differential scanning calorimetry (DSC). The related data are summarized in Table 1. In the thermogravimetric analysis (TGA) test, the 5% thermal decomposition temperature (*T*_d5%_) of the three polymers under the nitrogen atmosphere obtained from the curves (Figure 3) are all above 300°C, indicating that all the PBDT-IID polymers have good thermal stability. In addition, as the length of the OEG straight chain side chain increases, so does the polymer *T*_d5%_ value. The reason for this phenomenon is that the long OEG straight chains have better thermal stability compared to the short chains [25]. However, in the differential scanning calorimetry (DSC) tests, the three polymers (PBDT-IID2, PBDT-IID3, and PBDT-IID4) did not exhibit significant glass transition or phase change behavior, which was caused by the rigid structure of the polymer backbone and the strong interactions between the polymer molecular chains limiting the motion of the polymer molecular chain segments.

### 4.3. The Optical Properties of the PBDT-IID Polymers

**Figure 4:**
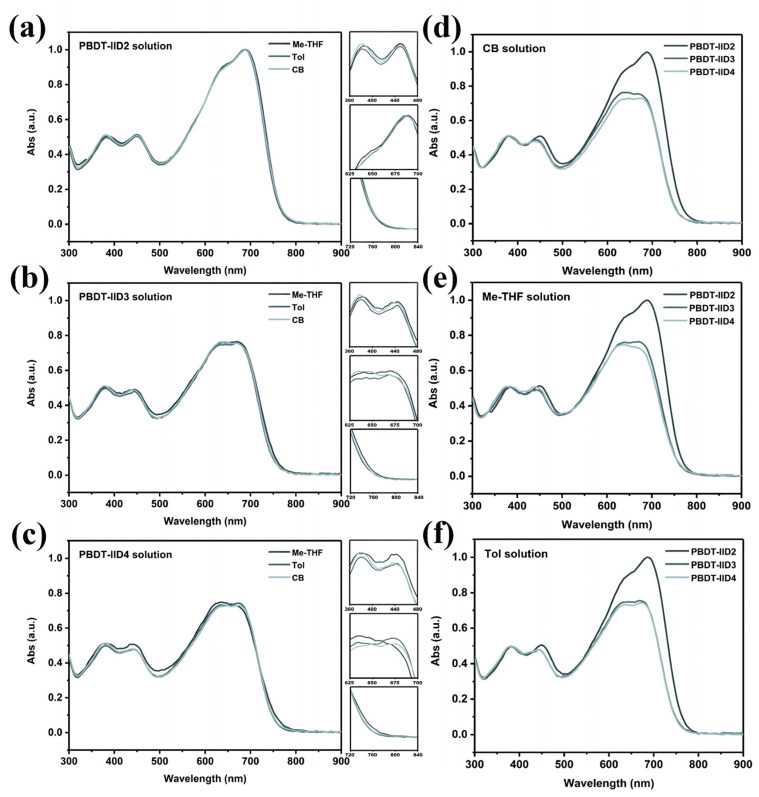
(**a**) UV-vis absorption spectra of PBDT-IID2 in Me-THF, toluene and CB at 25 °C, respectively; (**b**) UV-vis absorption spectra of PBDT-IID3 in Me-THF, toluene and CB at 25 °C, respectively; (**c**) UV-vis absorption spectra of PBDT-IID4 in Me-THF, toluene and CB at 25 °C, respectively; (**d**) UV-vis absorption spectra of PBDT-IID2, PBDT-IID3, and PBDT-IID4 in CB solution at 25 °C, respectively; (**e**) UV-vis absorption spectra of PBDT-IID2, PBDT-IID3, and PBDT-IID4 in Me-THF solution at 25 °C, respectively; (**f**) UV-vis absorption spectra of PBDT-IID2, PBDT-IID3, and PBDT-IID4 in toluene solution at 25 °C, respectively.

UV-vis absorption spectra of the polymers in a variety of organic solvents and film form have been acquired to understand the effect of different side chains on the optical properties of PBDT-IIDs. Figure 4a–f shows the UV-vis absorption spectra of the PBDT-IIDs dissolved in various solvents (chlorobenzene, toluene, and 2Me-THF) with different polarities. The results not surprisingly show that the UV-vis absorption spectra of the PBDT-IIDs in any solvent reveal two distinct characteristic absorption peaks. One of the high-energy absorption bands, located between 350–450 nm, is attributed to the π-π* transition of the BDT and IID units. The other low-energy broadband, located between 500 nm and 800 nm, is assigned to the charge transfer (CT) transition, which causes a significant redistribution of electrons along the backbone from the BDT unit to the acceptor unit. As illustrated in Figure 4a–f, all the polymers exhibit two peaks between 620 nm and 900 nm. The wavelengths and absorption intensities corresponding to these peaks (620–900 nm) of PBDT-IID2 are higher than those of PBDT-IID3 and PBDT-IID4 in the same solvent (Figure 4d–f), owing to the fact that shorter OEG side chains have smaller free volumes, and the smaller free volumes result in better intermolecular interactions for PBDT-IID2. In addition, the characteristic absorption peaks of the same polymer differ somewhat in various solvents (Figure 4a–c). Significantly due to the specific planar structure, organic conjugated materials strongly tend to form aggregates or supramolecular structures in solvents [26,27,28]. In previous studies, because of the different types of aggregates in solvents, their optical properties can be widely turned via the change of solubilizing substituents [29,30,31,32]. This phenomenon can be explained by the different types of aggregates of PBDT-IIDs containing various side chains (alkyl side chains and OEG side chains) in solvents with diverse polarities. Additionaly, the solution and solid film UV-vis absorption of the polymers PBDT-IID2, PBDT-IID3, and PBDT-IID4 in the same solvent were compared (Figure S2, taking THF as an example). The results showed that the characteristic absorption peak shapes of the solid film were similar to those of the solution state under the same solvent conditions, but the positions of the peaks were all red-shifted to some extent. This red-shift phenomenon is due to the interaction of the conjugated polymer backbone in the solid state, resulting in a better stacking state [33]. The onset absorption wavelengths of PBDT-IID2, PBDT-IID3, and PBDT-IID4 under solid-state films are 825 nm, 816 nm, and 811 nm, corresponding to the optical band gaps of 1.50 eV, 1.52 eV, and 1.53 eV, respectively. According to the previous literature, it is reasonable to assume that the main reason for the change in the optical properties of the PBDT-IIDs after changing the OEG side chain length may be that the increase in the length of the OEG side chain reduces the interaction between the polymer molecular chains and the inter-chains to a certain extent (increasing the polymer π-π accumulation distance), so the maximum characteristic absorption peak is slightly blue-shifted and the optical energy band gap is slightly improved (from 1.50 eV to 1.53 eV) [23]. The specific data differences and the optical band gaps calculated from the onset absorption wavelengths (solid film) are listed in Table 2.

### 4.4. The Electrochemical Properties of the PBDT-IID Polymers

**Figure 5:**
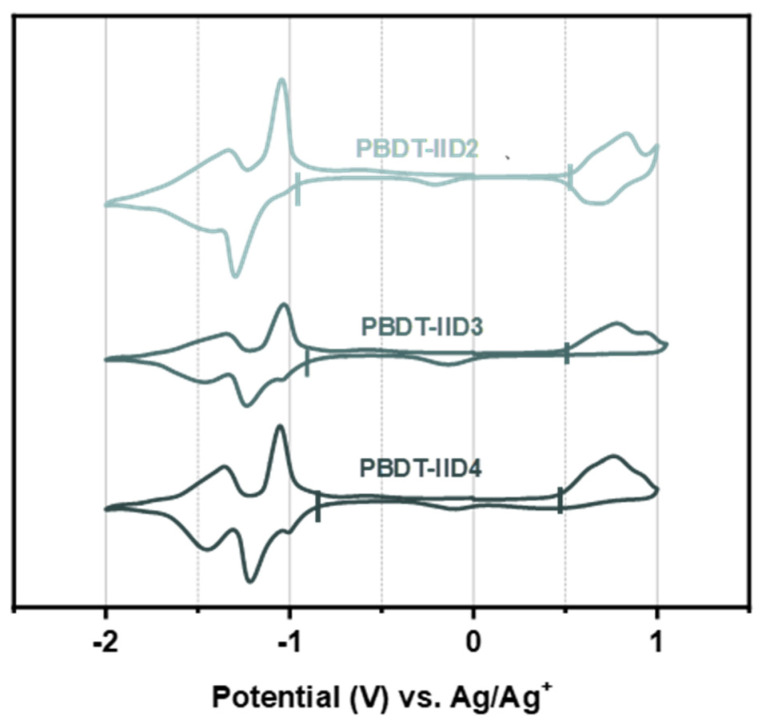
The cyclic voltammetric curves of PBDT-IID2, PBDT-IID3, and PBDT-IID4 (the electrolyte solution is 0.1 M tetrabutylammonium hexafluorophosphonate solution in anhydrous ACN).

The electrochemical properties of D-A conjugated polymers are one of the key factors affecting the performance of their corresponding optoelectronic devices; therefore, a preliminary work on the electrochemical properties of PBDT-IIDs polymers was carried out by cyclic voltammetry [34]. The measured cyclic voltammetry curves of the polymers PBDT-IID2, PBDT-IID3, and PBDT-IID4 are shown in Figure 5, and the relevant electrochemical data are summarized in Table 3. In the three-electrode system (working electrode: ITO conductive glass; reference electrode: Ag/Ag^+^ reference electrode; counter electrode: platinum wire electrode; internal standard: ferrocene Fe/Fe^+^; electrolyte solution: 1 mol/L acetonitrile solution of tetrabutylammonium hexafluorophosphonate), significant redox-peak pairs appeared for all three polymers under both forward and negative voltage scans. Among them, the redox peak pair appearing under the forward voltage scan was from the BDT unit, while the redox peak pair under the negative voltage came from the IID unit [18,19]. The onset oxidation potentials of the three polymers were 0.52 V (PBDT-IID2), 0.49 V (PBDT-IID3), and 0.45 V (PBDT-IID4). Compared with the onset oxidation/reduction potential ($E_{\mathrm{onset}}^{\mathrm{ox}}$/$E_{\mathrm{onset}}^{\mathrm{red}}$) and oxidation/reduction potential ($E_{1}^{\mathrm{ox}}$/$E_{2}^{\mathrm{red}}$) of PBDT-IID2, PBDT-IID3, and PBDT-IID4, they have lower onset oxidation/reduction potential ($E_{\mathrm{onset}}^{\mathrm{ox}}$/$E_{\mathrm{onset}}^{\mathrm{red}}$) and oxidation/reduction potential ($E_{1}^{\mathrm{ox}}$/$E_{2}^{\mathrm{red}}$), and show a trend that the onset oxidation potential of the polymers gradually decreases with the increasing length of the OEG linear side chains. We believe that there are two main reasons for this phenomenon. One is that OEG side chains are typical electron-giving groups, and the introduction of electron-giving groups undoubtedly decreases the onset oxidation potential of the materials [35,36]. Secondly, the increasing length of OEG side chains enhances the stacking distance between the molecular chains of PBDT-IIDs in the thin film state, which makes the injection and extraction of counter ions in the electrolyte easier when the electrochemical oxidation/reduction process occurs. In a further study, cyclic voltammetric curves of the polymers were tested with different electrolyte solvents or processing solvents (Figure S3), and the results showed that the patterns were all similar. From Equations (1) and (2), the HOMO/LUMO energy gaps of the three polymers can be calculated as 5.32 eV/3.78 eV (PBDT-IID2), 5.29 eV/3.73 eV (PBDT-IID3), and 5.25 eV/3.67 eV (PBDT-IID4) [37].

(1) $$E_{HOMO}=-e(E_{\mathrm{onset}}^{\mathrm{ox}}+4.8)$$

(2) $$E_{LUMO}=-e(E_{HOMO}-E_{g}^{\mathrm{opt}})$$

Based on the HOMO/LUMO energy level data of the PBDT-IIDs polymers, it can be found that the change of the OEG side chain length not only affects the solubility of the PBDT-IIDs in solvents but it also influences the HOMO/LUMO energy level of the PBDT-IIDs to a certain extent. Therefore, the introduction of OEG side chains into the main chain structures of D-A conjugated polymers can not only achieve the goal of green solvent processability of D-A conjugated polymers but also regulate the HOMO/LUMO energy levels of D-A conjugated polymers by changing the OEG side chain length, which provides a reference for the future molecular structure design of green solvent processable D-A conjugated polymers.

### 4.5. The Electrochromic Properties of the PBDT-IID Polymers

**Figure 6:**
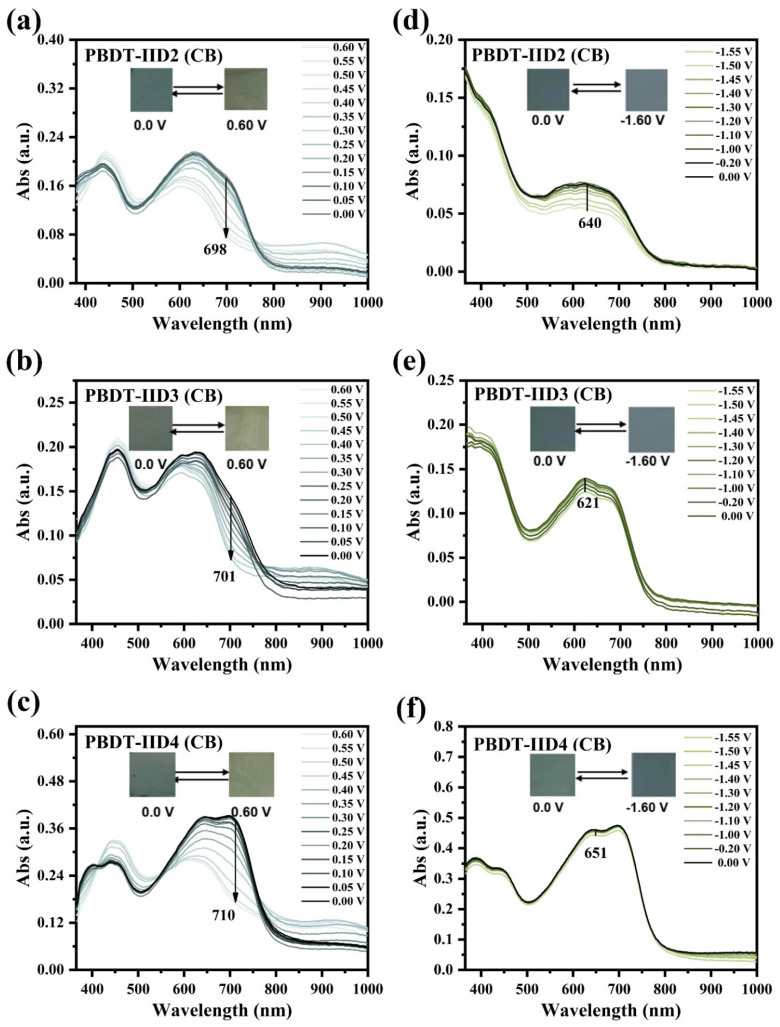
(**a**) UV-visible absorption spectra of PBDT-IID2 as a function of applied voltage (0–0.6 V); (**b**) UV-visible absorption spectra of PBDT-IID3 as a function of applied voltage (0–0.6 V); (**c**) UV-visible absorption spectra of PBDT-IID4 as a function of applied voltage (0–0.6 V); (**d**) UV-visible absorption spectra of PBDT-IID2 as a function of applied voltage (−1.6–0 V); (**e**) UV-visible absorption spectra of PBDT-IID3 as a function of applied voltage (−1.6–0 V); (**f**) UV-visible absorption spectra of PBDT-IID4 as a function of applied voltage (−1.6–0 V). The polymer films were processed using chlorobenzene and the test system was the same as the cyclic voltammetry test described above.

**Figure 7:**
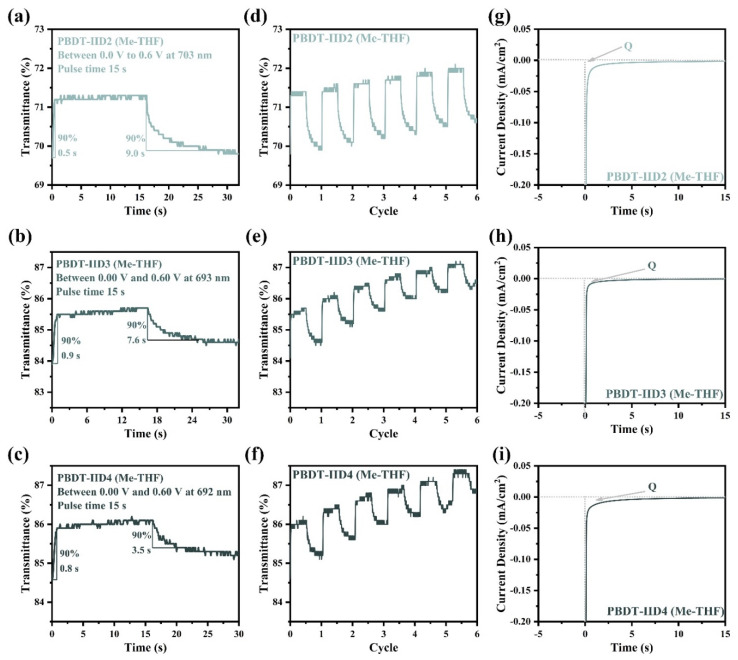
(**a**–**c**) The coloring/bleaching time and the optical contrast of PBDT-IID2, PBDT-IID3, and PBDT-IID4; (**d**–**f**) The variation of the optical contrast with the cycle number for PBDT-IID2, PBDT-IID3, and PBDT-IID4; (**g**–**i**) The current versus time curves of PBDT-IID2, PBDT-IID3, and PBDT-IID4 when coloration occurs. (The processing solvent for polymer films is Me-THF, Q represents the amount of charge injected or extracted per unit area.).

In the foregoing studies on the electrochemical properties of the PBDT-IIDs polymers, we found that all three polymers have redox peak pairs in both the forward potential scan (0–1.0 V) and the negative potential scan (−2.0–0 V). In accordance with the electrochemical doping principle, the PBDT-IIDs series polymers are capable of both P- and N-type doping under appropriate applied voltage conditions [38]. This implies that the polymers PBDT-IID2, PBDT-IID3, and PBDT-IID4 have the potential to undergo electrochromism at both positive and negative voltages, respectively, pending further experimental verification. Consequently, the suitability of PBDT-IIDs for electrochromic applications was initially determined by spectroelectrochemical testing, i.e., applying different constant voltages to polymer films spin-coated on ITO glass electrodes with a UV-vis spectrophotometer to detect the spectral absorption changes in the range of 400–1000 nm. For the purpose of forming a good ohmic contact between the PBDT-IIDs and the ITO glass electrodes (reducing the influence of electrodes on the electrochemical stability of PBDT-IIDs), PEDOT:PSS was used as an interfacial modification layer between the PBDT-IIDs and the ITO glass electrodes. Considering that the PEDOT component of the PEDOT:PSS is an electrochromic conjugated polymer with excellent properties, the spectroelectrochemical testing of PEDOT should be performed before the spectroelectrochemical testing of the PBDT-IIDs (Figure S4) [39]. The theoretical voltage range required for the spectroelectrochemical testing of the three polymers (negative: −1.6–0 V; positive: 0–0.6 V) was determined from the electrochemical properties study of PBDT-IIDs. The absorption changes in the UV-vis absorption spectra of the three polymers at different constant voltages after deducting the PEDOT: PSS are shown in Figure 6. During the scan with forward voltage (0–0.6 V), the intensity of the absorption spectra of the polymer films changed a little near the UV region and decreased significantly in the long wavelength visible region (near 700 nm) as the voltage gradually increased. This change appears macroscopically as a change in the film color from dark green in the neutral state to khaki in the oxidized state, which corresponds to the oxidation of the BDT units in the polymer structure. This reversible switching between yellow and green makes this type of material a clear prospect for application in even military camouflage [40,41,42]. The situation is different during the scan with negative potential (−1.6–0 V). With a gradual increase in the absolute value of the applied voltage, the intensity of the absorption spectrum of the polymer film does not change too much throughout the wavelength test range. However, this change can still be reflected macroscopically as an observable change in film color from dark green in the neutral state to light blue-green in the reduced state, corresponding to the reduction of IID units in the polymer structure. Nevertheless, as a whole, the change in the OEG side chain length has less effect on the spectroelectrochemical properties of PBDT-IID polymers. On the other hand, the effect of different processing solvents on the electrochromic properties of the polymers was also explored (Figure S5). The outcomes showed that the shapes of the absorption peaks of the polymer films with different processing solvents differed slightly, but the characteristic peak positions and intensity changes, the color changes, and the corresponding discoloration voltages were basically the same. This can be also supported by the color coordinates of polymer films in the neutral and oxidized states using different processing solvents (Table S2). This also confirmed the multi-selectivity of the processing solvents in the practical application of polymers. Afterwards, the films of PBDT-IID2, PBDT-IID3, and PBDT-IID4 were tested by the square wave potential method at the respective wavelengths with the largest change in absorption intensity to calculate the change in transmittance and coloring/bleaching time of the polymers during the electrochromic process (Figure 7a–c). For the term of this paper, the time to reach 90% of the maximum transmittance change is defined as the coloring/bleaching time. The coloring time of the PBDT-IIDs is extremely short (less than 1 s) under forward voltage scanning (0.5 s, 0.9 s, and 0.8 s for PBDT-IID2, PBDT-IID3, and PBDT-IID4, respectively) from the test results, while the corresponding bleaching progress needs to take several seconds (9.0 s, 7.6 s, and 3.5 s for PBDT-IID2, PBDT-IID3 and PBDT-IID4, seperately) to complete, which is consistent with the coloring/bleaching time pattern of the conjugated polymers [43,44]. Another reason for the significantly shorter coloring times for PBDT-IIDs compared to other conjugated polymers than the bleaching time is the introduction of the side chain OEG. It enhances the interaction between the polymer and the electrolyte and causes it to be more favorable for complementary ion doping [37]. In the negative voltage scan, the coloring time of the PBDT-IIDs is less than two seconds, although the transmittance change of the polymer is smaller than that in the positive direction. In fact, the optical contrast change (ΔT) of all the three mention above under negative voltage is less than 0.1%, which is far below the minimum standard (ΔT ≥ 30%) for the application of conjugated polymers in the electrochromic field. Hence, PBDT-IIDs as N-doped CPs cannot be directly applied in the domain of electrochromic applications, but considering that PBDT-IIDs can change from dark green to light blue-green under negative voltage, it is obvious that PBDT-IIDs can be used as a class of green tunable electrochromic materials to be mixed with other electrochromic materials according to the RGB triplet superposition theory to gain a wide color gamut electrochromic material [45]. The coloring efficiency of the three polymers processed with Me-THF was calculated from the equation: η = ΔOD/Q (where ΔOD is the optical density, calculated from the transmittance curve, and Q is the charge density, calculated from the current-time curve (Figure 7g–i)) to be 213.10 mC/cm^2^, 214.12 mC/cm^2^, and 242.06 mC/cm^2^, respectively. The same tests were performed on polymer films processed with other solvents, and similar results were obtained (Figures S6 and S7 and Table S3). Since the transmittance change of the film decays rapidly during the test (Figure 7d–f), the cyclic stability is not discussed too much here. The detailed electrochromic data are summarized in Table 4.

## 5. Conclusions

A series of D-A alternating conjugated polymers (PBDT-IID2, PBDT-IID3, and PBDT-IID4) containing different lengths of OEG side chains were prepared by molecular modification of isoindocyanine with OEG side chains followed by a Stille coupling reaction with benzodithiophene. The introduction of OEG side chains with many advantages, such as high hydrophilicity, high polarity, high flexibility, and good biocompatibility, has improved the overall performance of the PBDT-IIDs to some extent, especially their solubility in polar non-halogenated benzene-free solvents, which brings great convenience to the subsequent processing. This can ultimately be attributed to the effect of the OEG side chain length on the degree of molecular chain stacking in its thin film state. The electrochromic behavior of the PBDT-IIDs under both positive and negative voltages makes them have excellent advantages for practical applications. To expand, the color transition of the PBDT-IIDs between the neutral (dark green) and oxidized (khaki) states is undoubtedly an excellent candidate for military camouflage; and the transition between the neutral (dark green) and reduced (light blue-green) states allows it to provide green (one of the three primary RGB colors) in electrochromic device preparation. In conclusion, the design strategy can provide a reference for a feasible green solution for subsequent material development.

## Figures and Tables

**Table 1:** The molecular weights and thermal stabilities of PBDT-IIDs.

Polymer	GPC (kDa) ^[a]^			Thermal Stabilities (°C)
	*M*_n_	*M*_w_	Đ	*T*_d5%_ ^[b]^
**PBDT-IID2**	54	118.8	2.20	314.7
**PBDT-IID3**	49	179.8	3.67	318.0
**PBDT-IID4**	37	181.3	4.91	318.5

^[a]^: The *M*_n_, *M*_w,_ and dispersity (*Đ*) were confirmed by GPC in CH_2_Cl_2_ using polystyrene as a standard reference. ^[b]^: Recorded by TGA at a heating rate of 10 °C/min in N_2_.

**Table 2:** The optical properties of the PBDT-IID solutions (in THF) and thin films.

Polymer		PBDT-IID2	PBDT-IID3	PBDT-IID4
**Solution ^[a]^**	BDT π-π*	380	378	377
	IID π-π*	450	445	444
	*λ*_max_ (nm)	687	672	669
**Film ^[b]^**	BDT π-π*	391	388	385
	IID π-π*	452	447	445
	*λ*_max_ (nm)	702	696	692
	*λ*_onset_ (nm)	807	793	787
	$E_{g}^{\mathrm{opt}}$ (eV)	1.54	1.56	1.58

^[a]^ The concentration was 1 × 10^−5^ M in THF. ^[b]^ $E_{g}^{\mathrm{opt}}$ = 1240/*λ*_onset_.

**Table 3:** The electrochemical properties of the PBDT-IIDs.

Polymer	Oxidation (V)			Reduction (V)			Energy Level (eV)	
	$E_{\mathrm{onset}}^{\mathrm{ox}}$	$E_{1}^{\mathrm{ox}}$	$E_{2}^{\mathrm{ox}}$	$E_{\mathrm{onset}}^{\mathrm{red}}$	$E_{1}^{\mathrm{red}}$	$E_{2}^{\mathrm{red}}$	HOMO	LUMO
**PBDT-IID2**	0.52	0.84	−1.04	−0.88	0.67	−1.30	5.32	3.78
**PBDT-IID3**	0.49	0.78	−1.03	−0.84	--	−1.24	5.29	3.73
**PBDT-IID4**	0.45	0.76	−1.04	−0.82	--	−1.21	5.25	3.67

**Table 4:** The electrochromic properties of the PBDT-IIDs.

Solvents	Polymers	ΔT (%)	Response Time (s) t_c_/t_b_	CE (cm^2^/C)
**Me-THF**	**PBDT-IID2**	1.53	0.5 s/9.0 s	213.10
	**PBDT-IID3**	1.46	0.9 s/7.6 s	214.12
	**PBDT-IID4**	1.51	0.8 s/3.5 s	242.06

## Supplementary Materials

The following supporting information can be downloaded at: https://www.mdpi.com/article/10.3390/ma16010060/s1, Figure S1: NMR spectra of monomers and polymers; Figure S2: Comparison of UV-visible absorption spectra of PBDT-IID2, PBDT-IID3 and PBDT-IID4 in solution and films (the solvent is THF); Figure S3: (a–c) Cyclic voltammetric curves of PBDT-IID2, PBDT-IID3 and PBDT-IID4 (electrolyte solution is 0.1M tetrabutylammonium hexafluorophosphonate solution in anhydrous propylene carbonate, the solvents used for the preparation of the films are toluene, chlorobenzene and methyltetrahydrofuran, respectively); (d) Cyclic voltammetric curves of ITO and ferrocene in propylene carbonate electrolyte; Figure S4: UV-vis absorption spectra of PEDOT films at different applied voltages; Figure S5: (a–c) UV-visible absorption spectra of PBDT-IID2, PBDT-IID3, and PBDT-IID4 as a function of applied voltage (0–0.6 V, the processing solution is toluene); (d–f) UV-visible absorption spectra of PBDT-IID2, PBDT-IID3, and PBDT-IID4 as a function of applied voltage (0–0.6 V, the processing solution is methyl tetrahydrofuran); (g–i) UV-visible absorption spectra of PBDT-IID2, PBDT-IID3, and PBDT-IID4 as a function of applied voltage (−1.6–0 V, the processing solution is toluene); (j–l) UV-visible absorption spectra of PBDT-IID2, PBDT-IID3, and PBDT-IID4 as a function of applied voltage (−1.6–0 V, the processing solution is methyl tetrahydrofuran). Figure S6: (a–c) Coloring/bleaching time and optical contrast of PBDT-IID2, PBDT-IID3 and PBDT-IID4; (d–f) Variation of optical contrast with cycle number for PBDT-IID2, PBDT-IID3 and PBDT-IID4; (g–i) Current versus time curves of PBDT-IID2, PBDT-IID3 and PBDT-IID4 when coloration occurs; Figure S7: (a–c) Coloring/bleaching time and optical contrast of PBDT-IID2, PBDT-IID3 and PBDT-IID4; (d–f) Variation of optical contrast with cycle number for PBDT-IID2, PBDT-IID3 and PBDT-IID4; (g–i) Current versus time curves of PBDT-IID2, PBDT-IID3 and PBDT-IID4 when coloration occurs; Table S1: Solubility of PBDT-IIDs in non-halogen solvents; Table S2: Color coordinates of PBDT-IIDs films in its neutral and oxidized states using different processing solvents; Table S3: Detailed electrochromic data of PBDT-IIDs under different process solvents.
