# Supplementary material for: Preparation and Electrochromic Properties of Benzodithiophene-Isoindigo Conjugated Polymers with Oligoethylene Glycol Side Chains
Source: Materials (Basel). 2022 Dec 21;16(1):60. doi: 10.3390/ma16010060 (PMC9821313; doi:10.3390/ma16010060)
Supplement: Supplementary file 1 [file materials-16-00060-s001.zip › materials-2054562-supplementary.pdf]

Supporting Information

Preparation and Electrochromic Properties of Benzodithiophene-Isoindigo Conjugated Polymers with Oligoethylene Glycol Side Chains

Qilin Wang [1], Yuehui Zhai [2], Danming Chao [1], Zheng Chen [1,*] and Zhenhua Jiang [1]

[1] Engineering Research Center of Special Engineering Plastics, Ministry of Education, National and Local Joint Engineering Laboratory for Synthetic Technology of High Performance Polymer, College of Chemistry, Jilin University, Changchun 130012, China

[2] School of Materials and Energy, University of Electronic Science and Technology of China, Chengdu 611731, China

* Correspondence: chenzheng2013@jlu.edu.cn.

Table of Contents

1. NMR spectra of the monomers and polymers. 3-10

2. Comparison of UV-visible absorption spectra 11

3. Cyclic voltammetric curves 12

4. UV-visible absorption spectra 13

5. Electrochromic properties of PBDT-IIDs under different process solvents 14-16

6. Solubility of PBDT-IIDs 17

7 Color coordinates of PBDT-IIDs films 17

8. Electrochromic data of PBDT-IIDs under different process solvents. 17

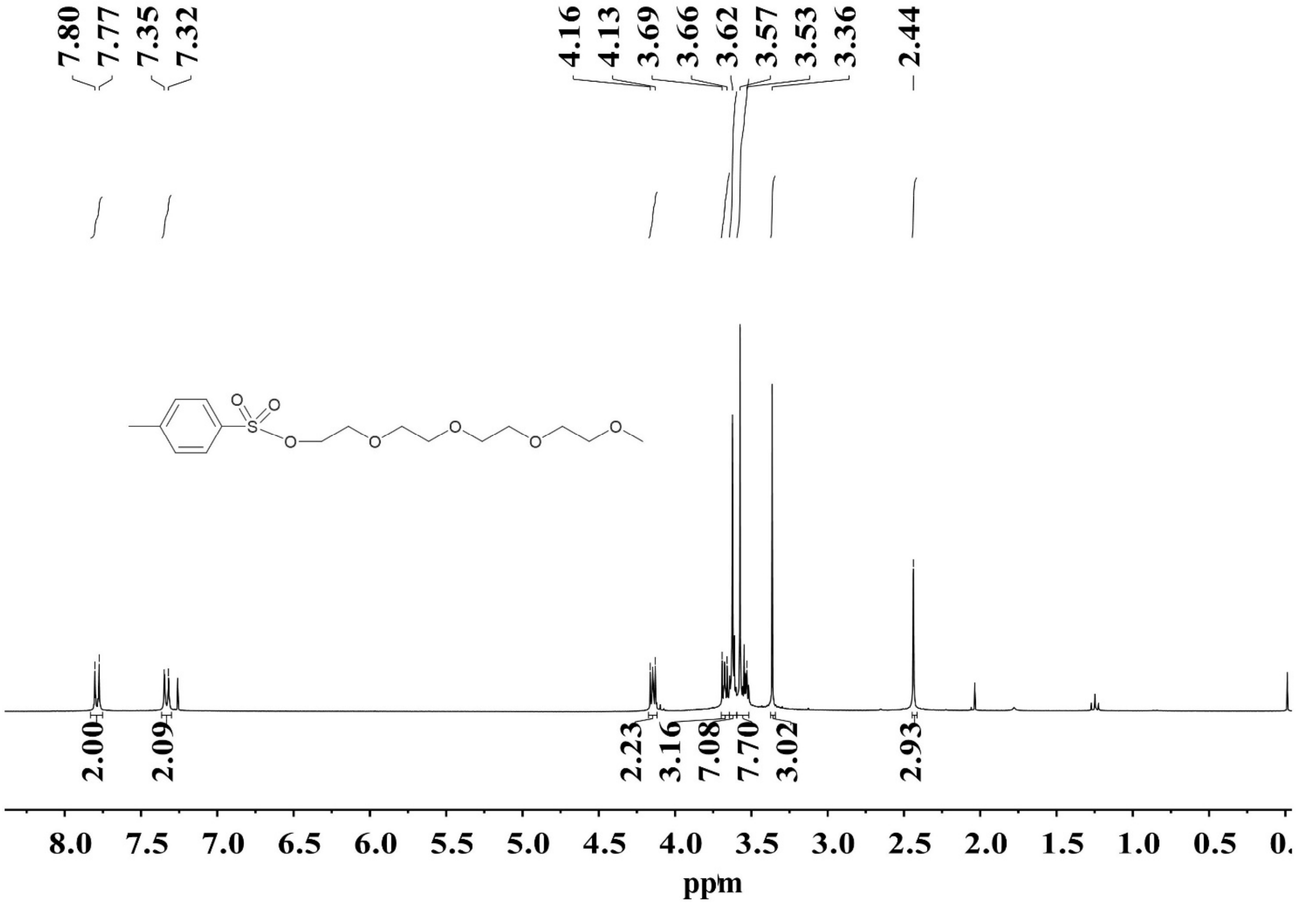
7.80
7.77
7.35
7.32
4.16
4.13
3.69
3.66
3.62
3.57
3.53
3.36
2.44
2.00
2.09
2.23
3.16
7.08
7.70
3.02
2.93
8.0
7.5
7.0
6.5
6.0
5.5
5.0
4.5
4.0
3.5
3.0
2.5
2.0
1.5
1.0
0.5
ppm

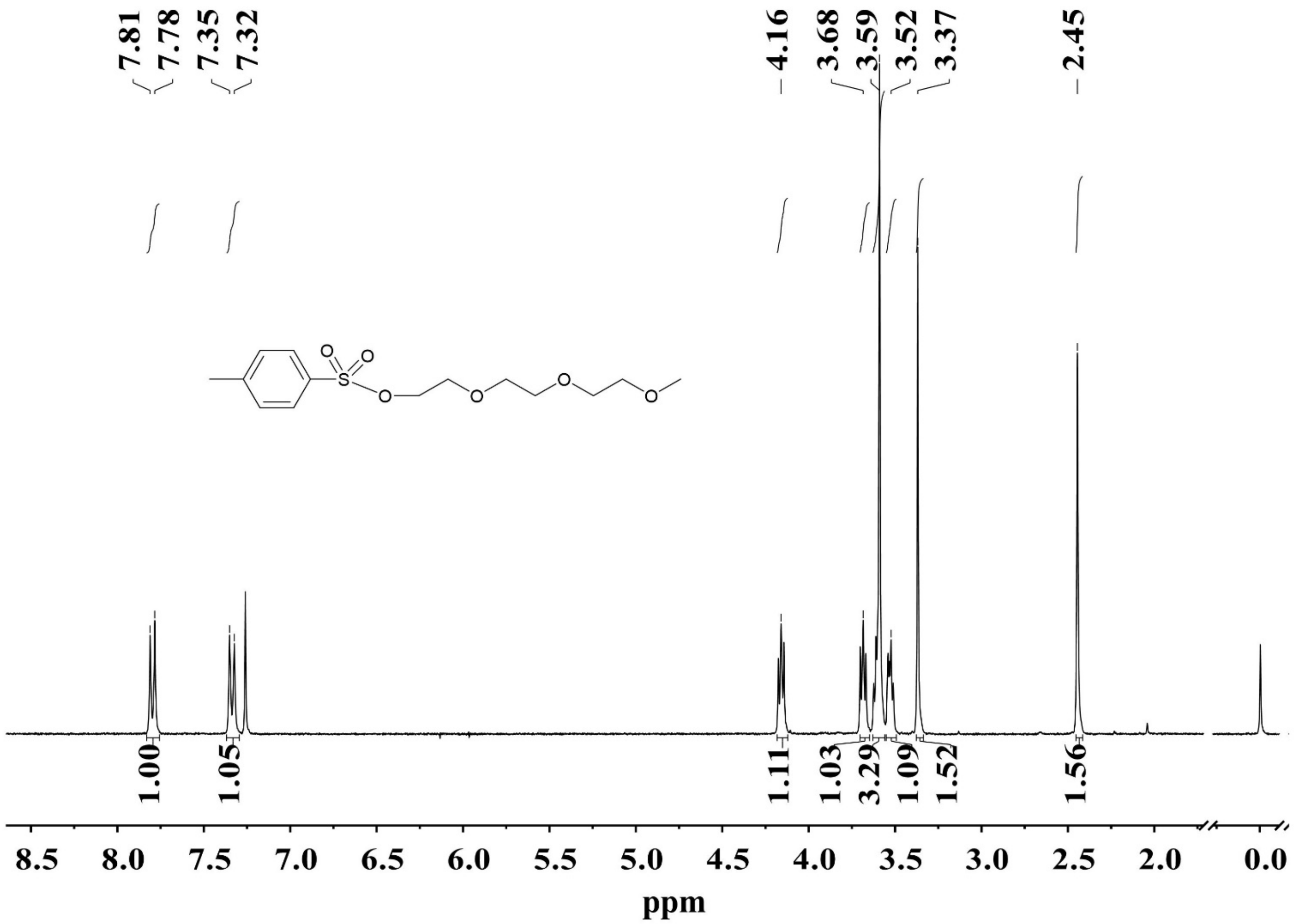
7.81
7.78
7.35
7.32
4.16
3.68
3.59
3.52
3.37
2.45
1.00
1.05
1.11
1.03
3.29
1.09
1.52
1.56
8.5 8.0 7.5 7.0 6.5 6.0 5.5 5.0 4.5 4.0 3.5 3.0 2.5 2.0 0.0
ppm
O
S
O
O
O
O
O

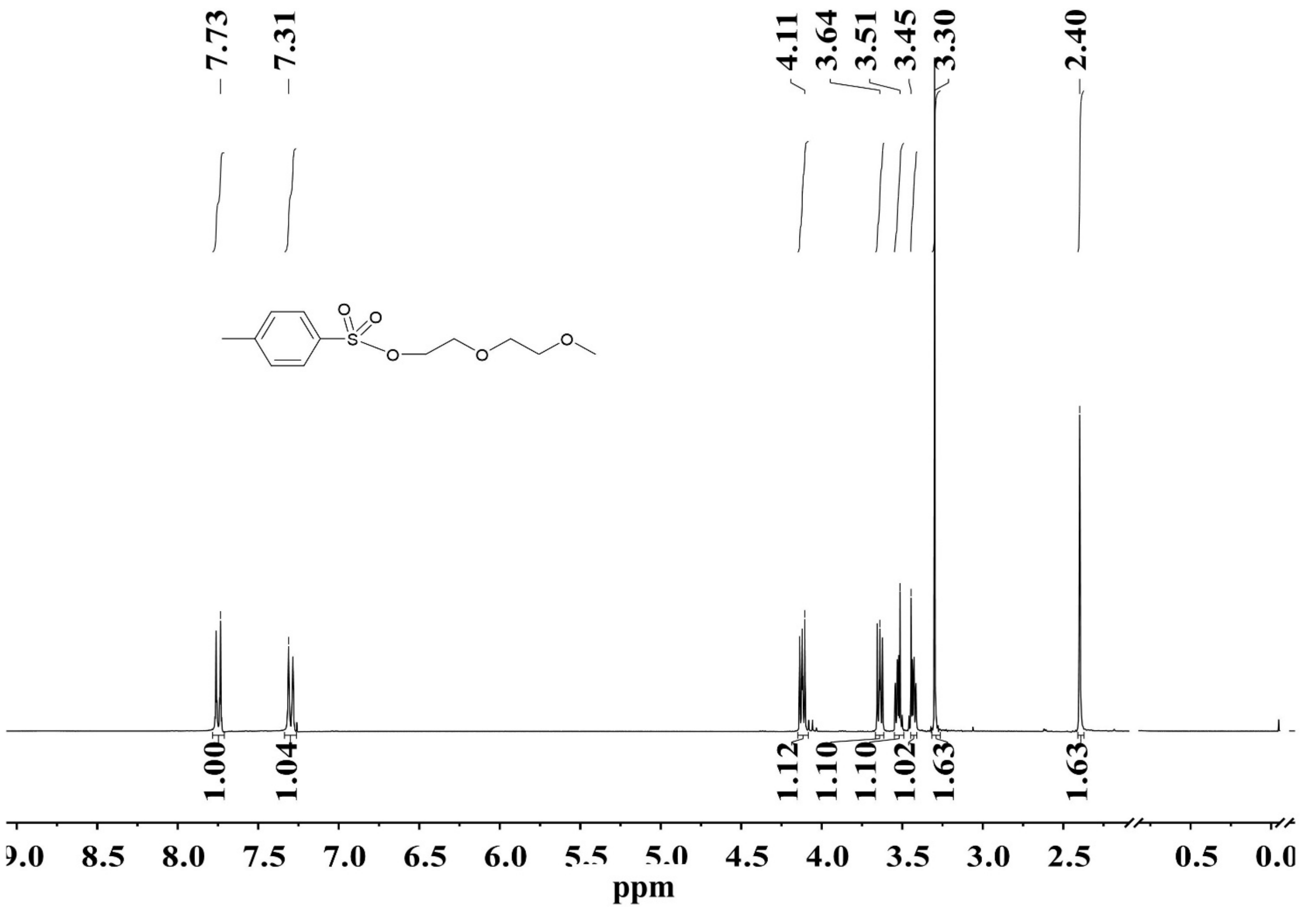
7.73
7.31
4.11
3.64
3.51
3.45
3.30
2.40
1.00
1.04
1.12
1.10
1.10
1.02
1.63
1.63
8.5
8.0
7.5
7.0
6.5
6.0
5.5
5.0
4.5
4.0
3.5
3.0
2.5
0.5
ppm

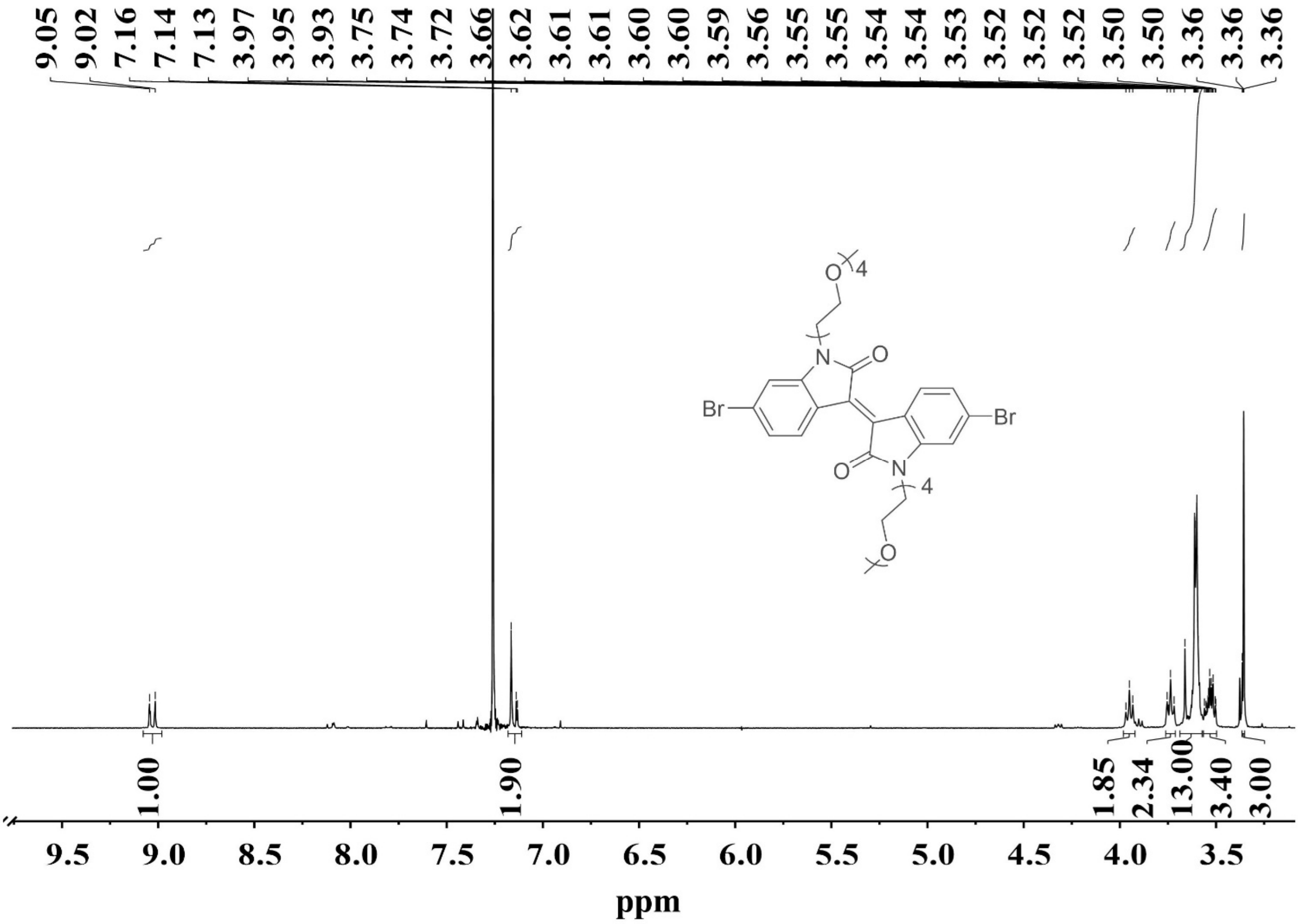
9.05
9.02
7.16
7.14
7.13
3.97
3.95
3.93
3.75
3.74
3.72
3.66
3.62
3.61
3.61
3.60
3.60
3.59
3.56
3.55
3.55
3.54
3.54
3.53
3.52
3.52
3.52
3.50
3.50
3.36
3.36
3.36
Br
N
O
O
4
Br
N
O
O
4
1.00
1.90
1.85
2.34
13.00
3.40
3.00
9.5
9.0
8.5
8.0
7.5
7.0
6.5
6.0
5.5
5.0
4.5
4.0
3.5
ppm

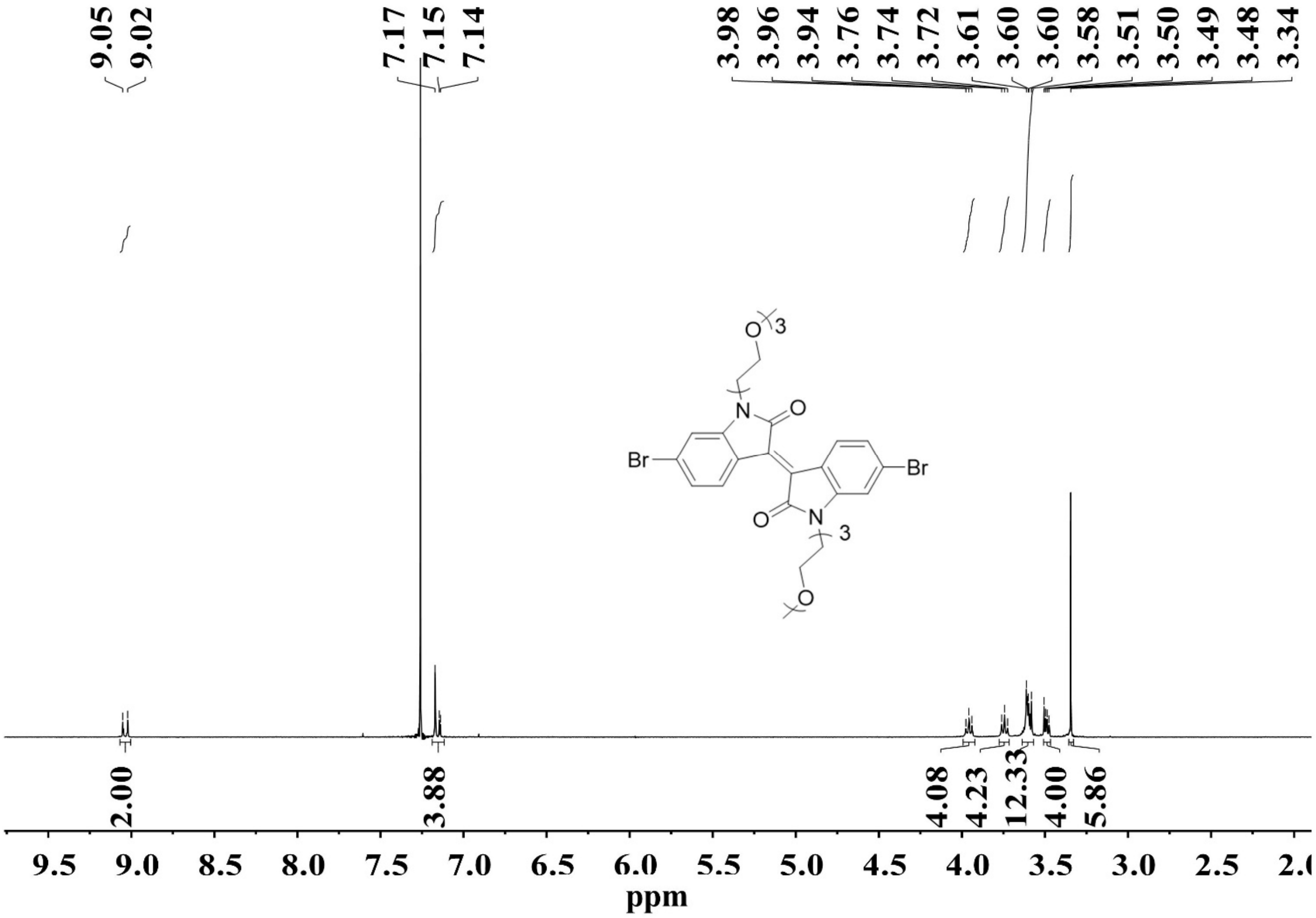
9.05
9.02
7.17
7.15
7.14
3.98
3.96
3.94
3.76
3.74
3.72
3.61
3.60
3.60
3.58
3.51
3.50
3.49
3.48
3.34
Br
Br
N
N
O
O
O
O
3
3
2.00
3.88
4.08
4.23
12.33
4.00
5.86
9.5
9.0
8.5
8.0
7.5
7.0
6.5
6.0
5.5
5.0
4.5
4.0
3.5
3.0
2.5
ppm

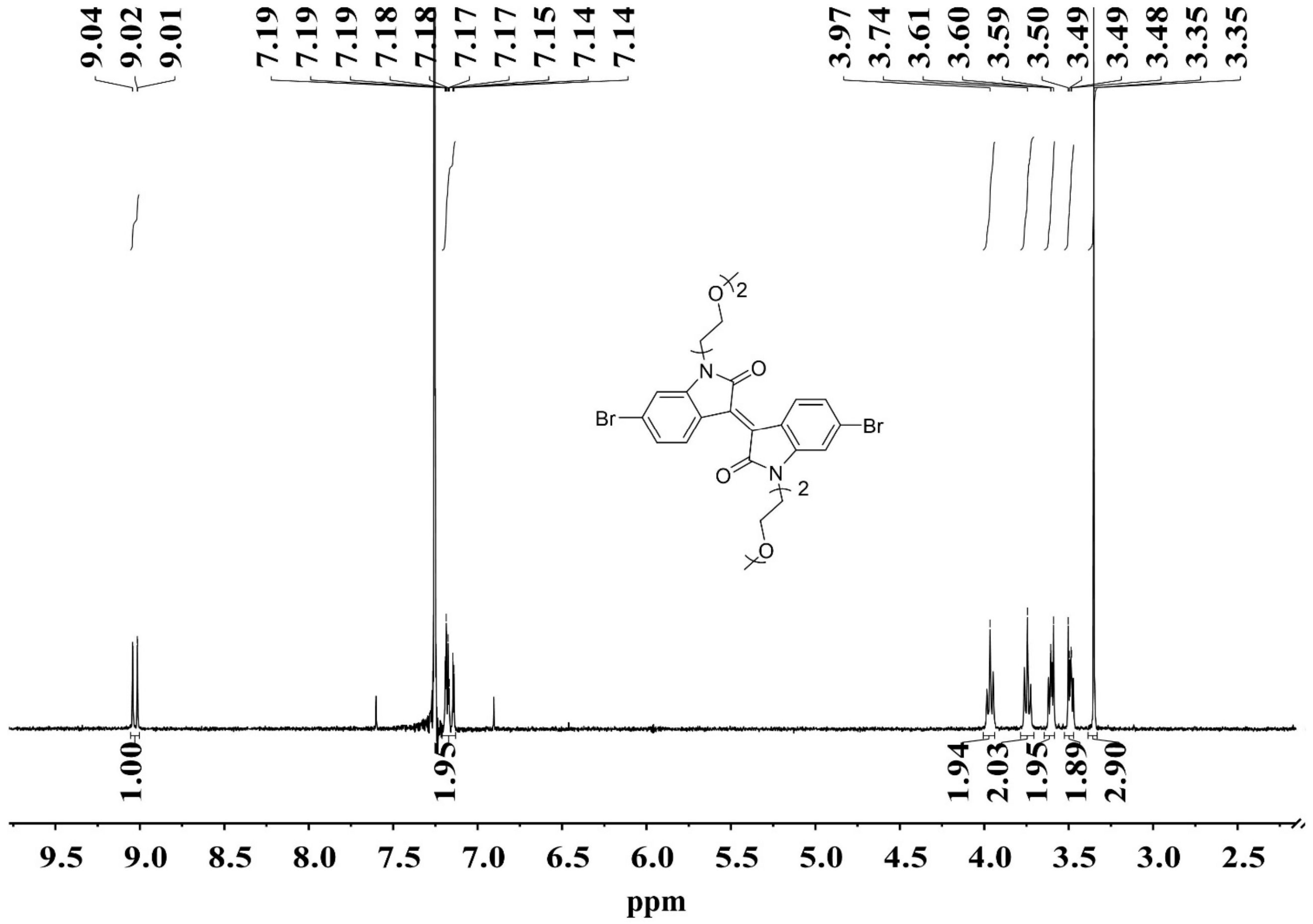
9.04
9.02
9.01
7.19
7.19
7.19
7.18
7.18
7.17
7.17
7.15
7.14
7.14
3.97
3.74
3.61
3.60
3.59
3.50
3.49
3.49
3.48
3.35
3.35
O
2
N
O
Br
Br
O
N
2
O
1.00
1.95
1.94
2.03
1.95
1.89
2.90
9.5 9.0 8.5 8.0 7.5 7.0 6.5 6.0 5.5 5.0 4.5 4.0 3.5 3.0 2.5
ppm

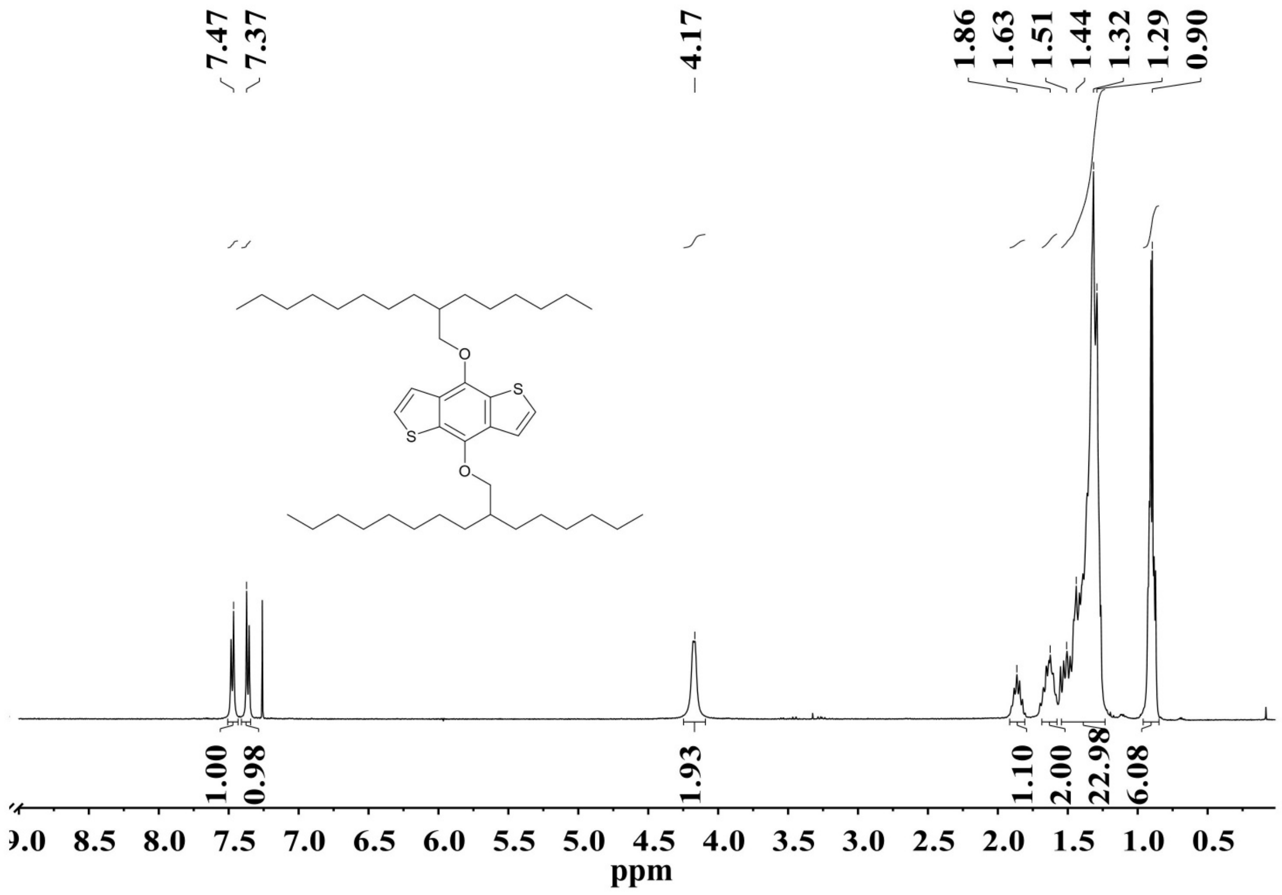
7.47
7.37
4.17
1.86
1.63
1.51
1.44
1.32
1.29
0.90
1.00
0.98
1.93
1.10
2.00
22.98
6.08
9.0 8.5 8.0 7.5 7.0 6.5 6.0 5.5 5.0 4.5 4.0 3.5 3.0 2.5 2.0 1.5 1.0 0.5
ppm
O
S
S
O

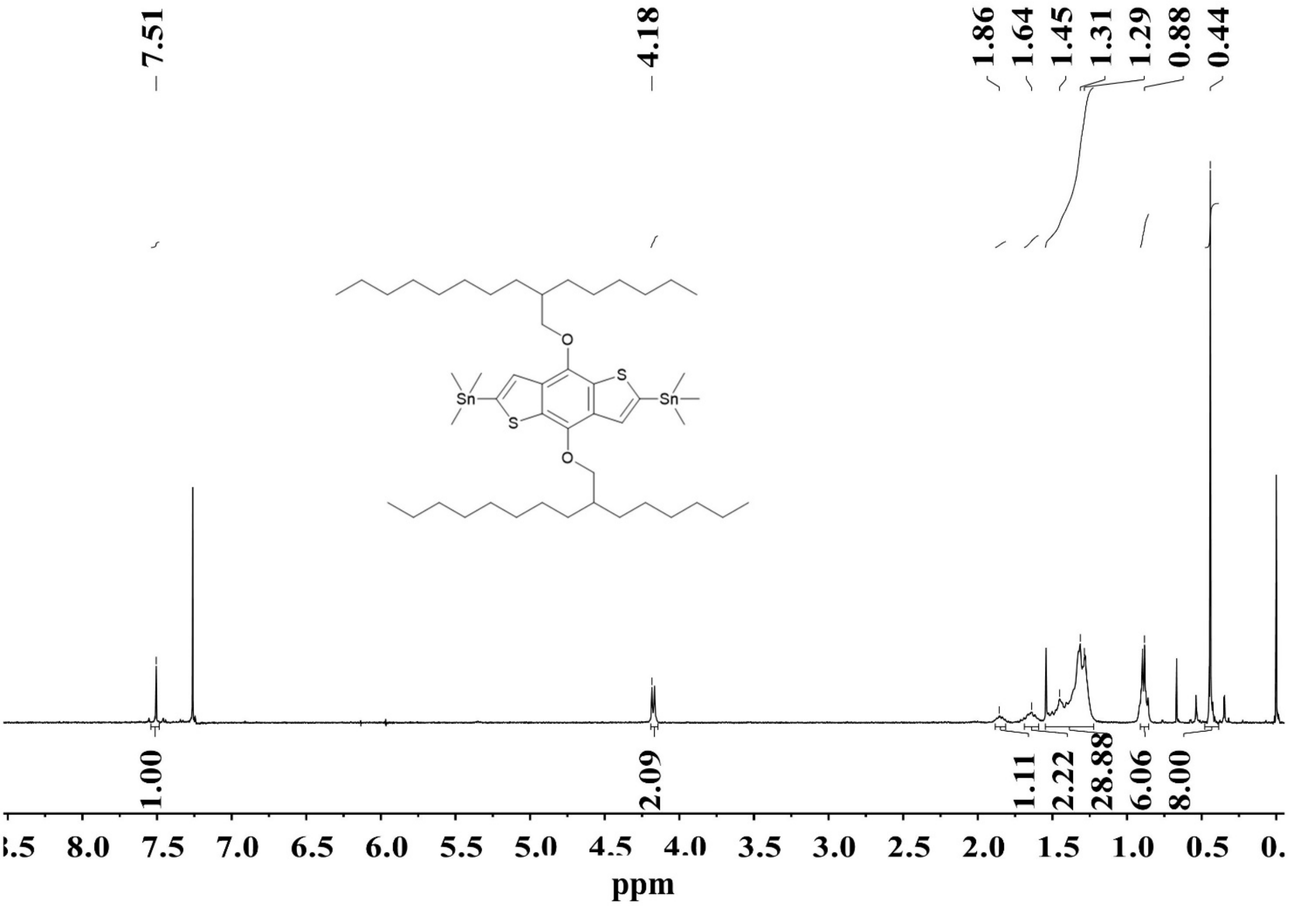
7.51
4.18
1.86
1.64
1.45
1.31
1.29
0.88
0.44
Sn
S
O
1.00
2.09
1.11
2.22
28.88
6.06
8.00
8.0 7.5 7.0 6.5 6.0 5.5 5.0 4.5 4.0 3.5 3.0 2.5 2.0 1.5 1.0 0.5
ppm

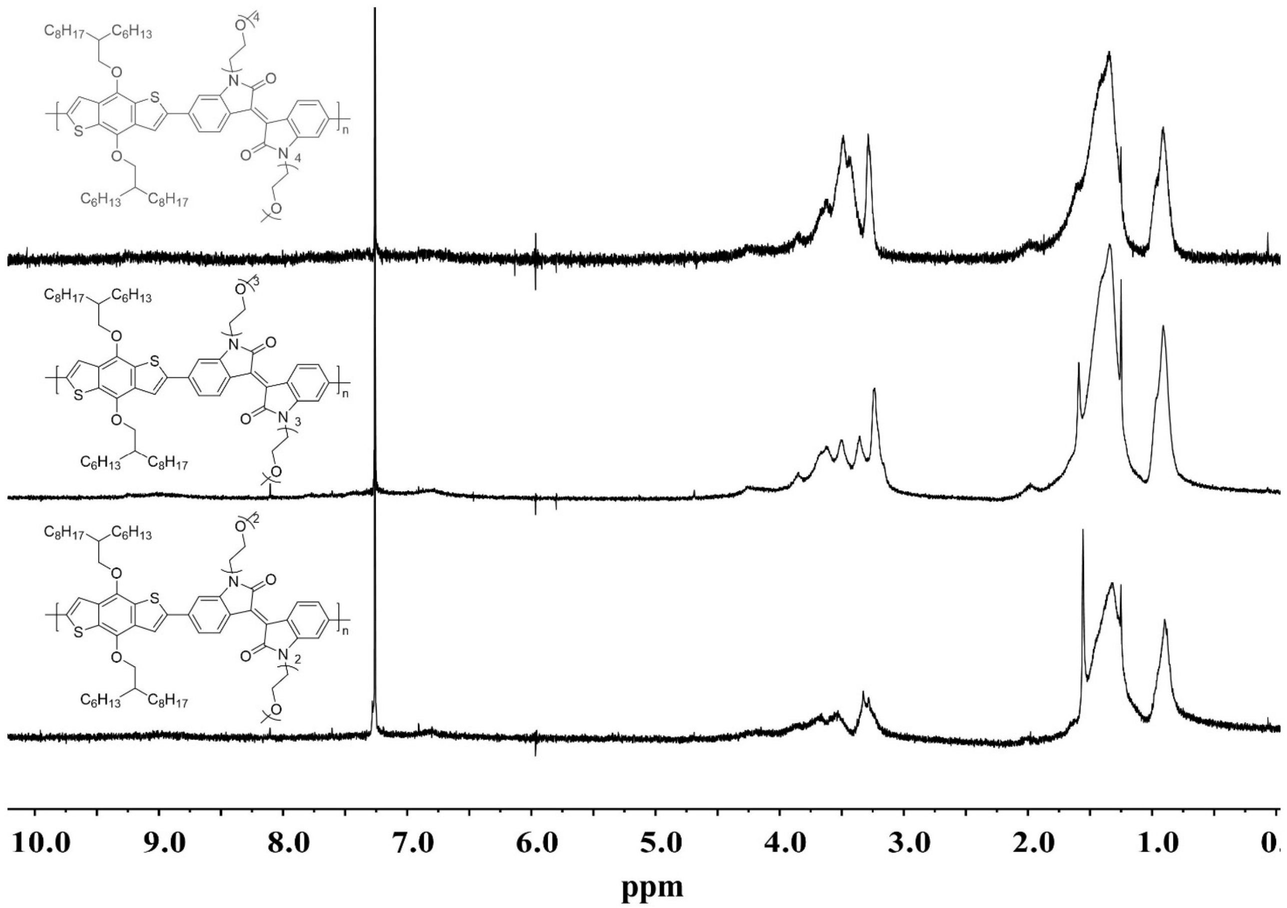

Figure S1. NMR spectra of monomers and polymers.

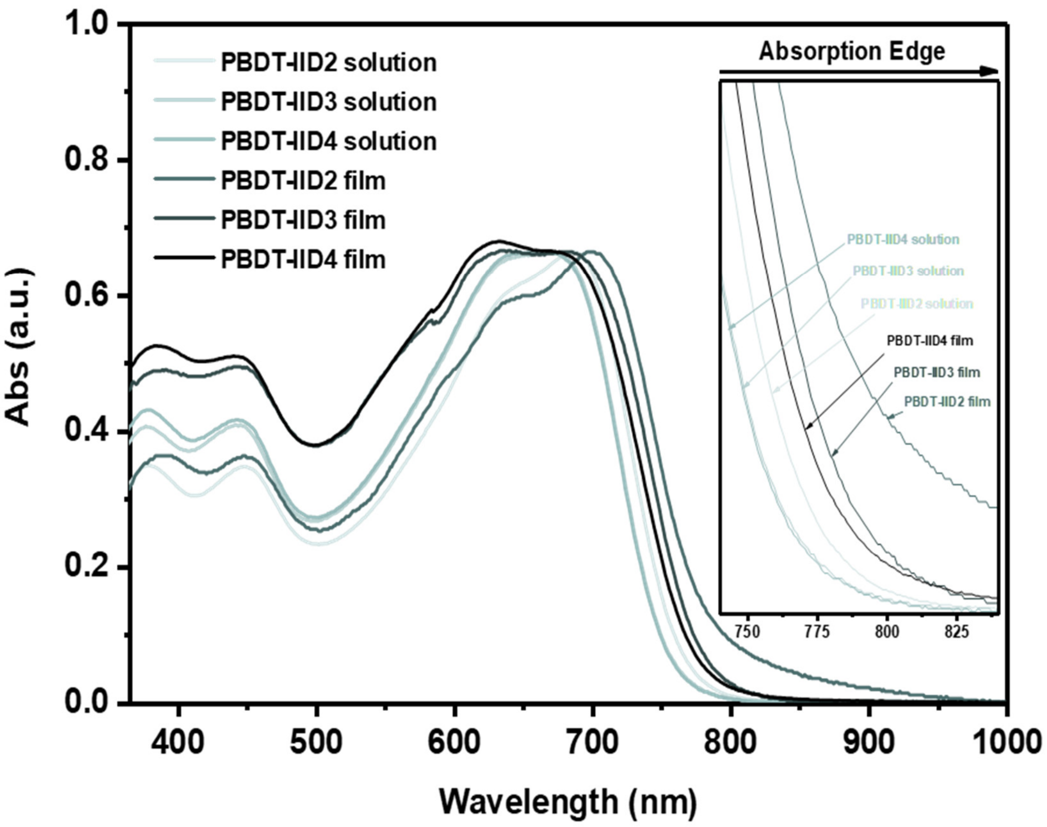

Figure S2. Comparison of UV-visible absorption spectra of PBDT-IID2, PBDT-IID3 and PBDT-IID4 in solution and films (the solvent is THF).

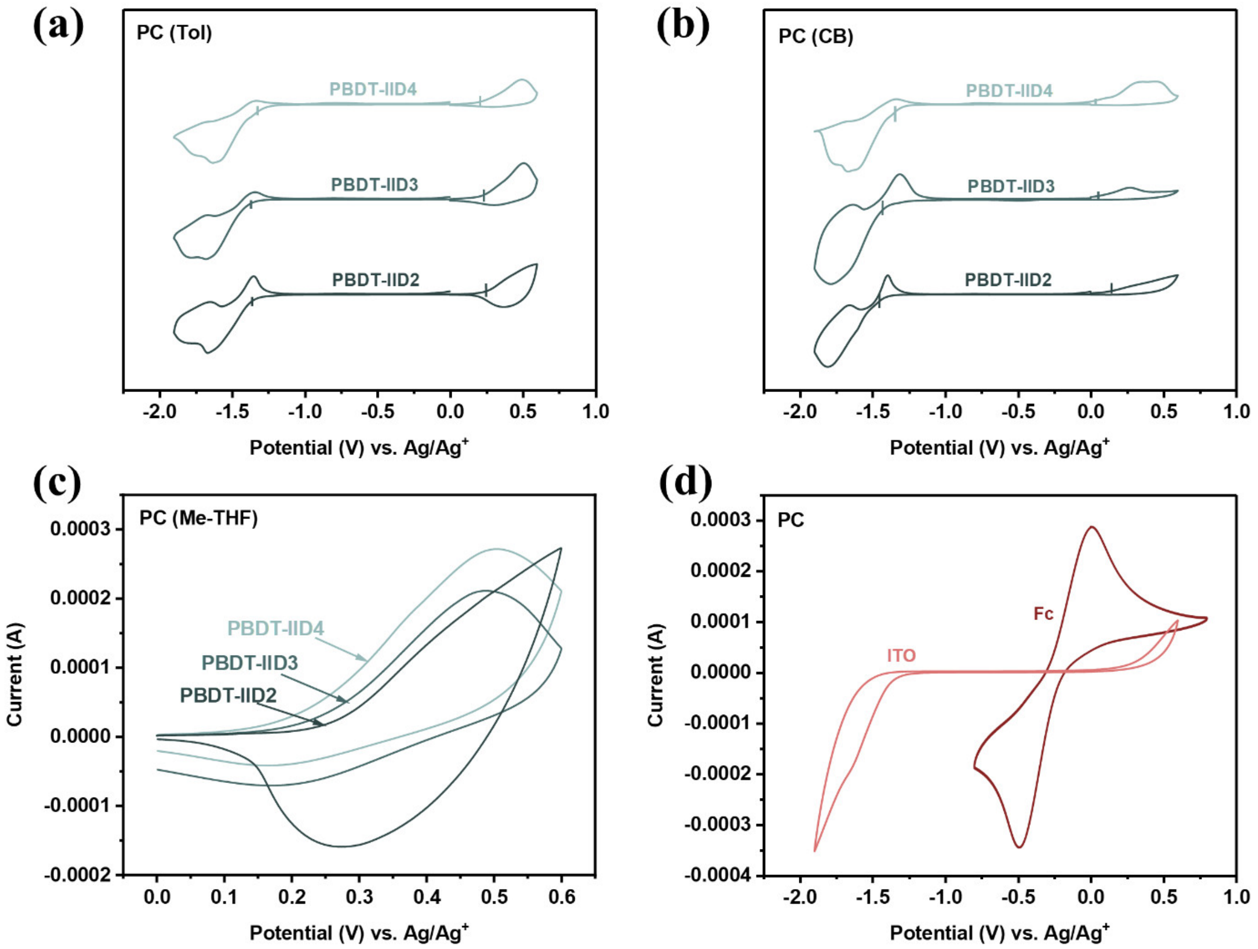

Figure S3. (a–c) Cyclic voltammetric curves of PBDT-IID2, PBDT-IID3 and PBDT-IID4 (electrolyte solution is 0.1M tetrabutylammonium hexafluorophosphonate solution in anhydrous propylene carbonate, the solvents used for the preparation of the films are toluene, chlorobenzene and methyltetrahydrofuran, respectively); (d) Cyclic voltammetric curves of ITO and ferrocene in propylene carbonate electrolyte.

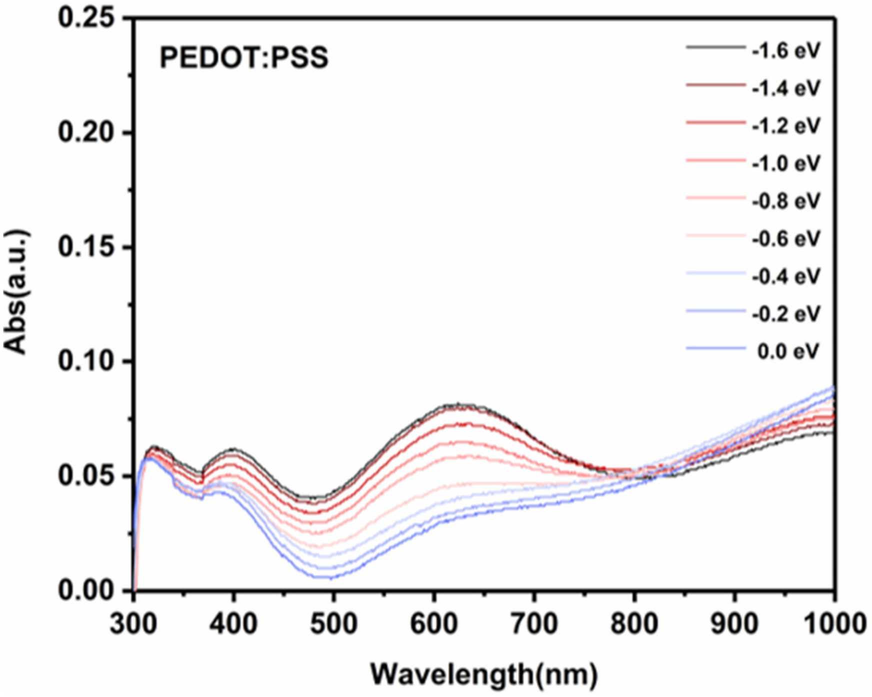

Figure S4. UV-vis absorption spectra of PEDOT films at different applied voltages.

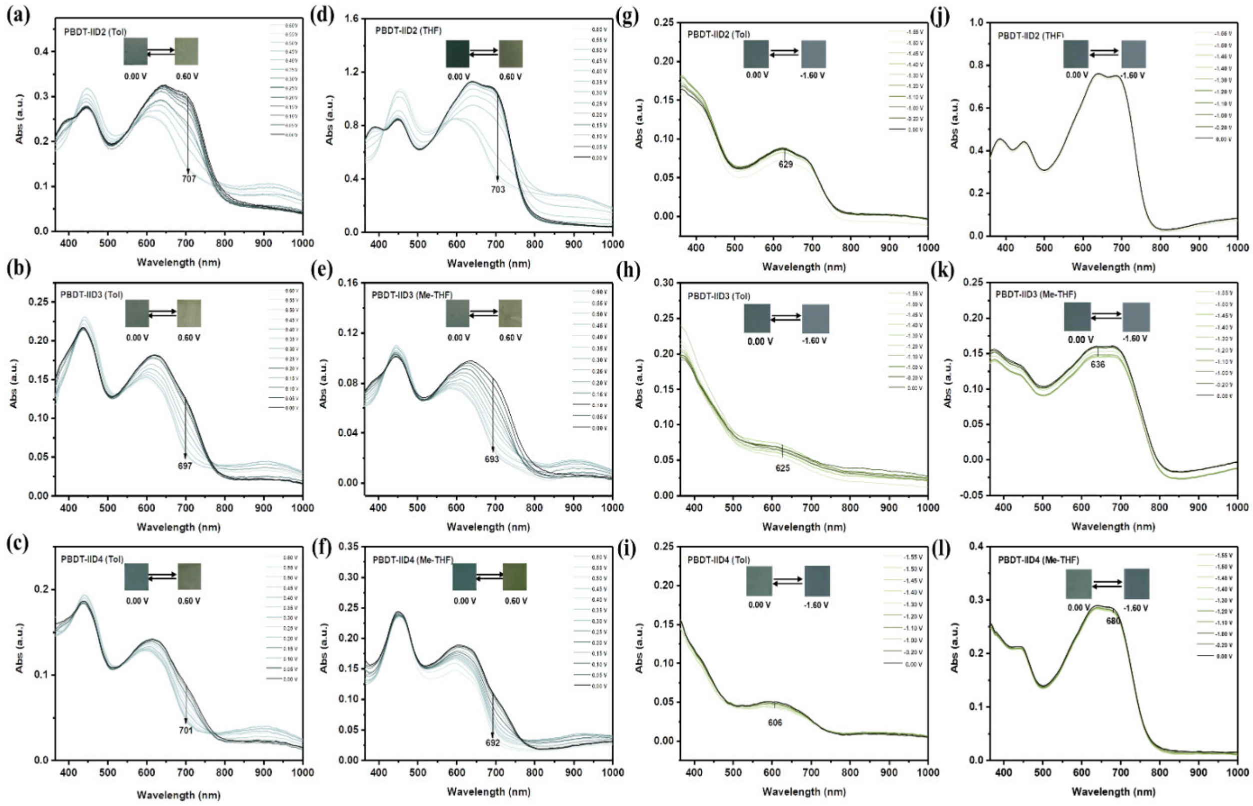

Figure S5. (a–c) UV-visible absorption spectra of PBDT-IID2, PBDT-IID3, and PBDT-IID4 as a function of applied voltage (0–0.6 V, the processing solution is toluene); (d–f) UV-visible absorption spectra of PBDT-IID2, PBDT-IID3, and PBDT-IID4 as a function of applied voltage (0–0.6 V, the processing solution is methyl tetrahydrofuran); (g–i). UV-visible absorption spectra of PBDT-IID2, PBDT-IID3, and PBDT-IID4 as a function of applied voltage (−1.6–0 V, the processing solution is toluene); (j–l). UV-visible absorption spectra of PBDT-IID2, PBDT-IID3, and PBDT-IID4 as a function of applied voltage (−1.6–0 V, the processing solution is methyl tetrahydrofuran).

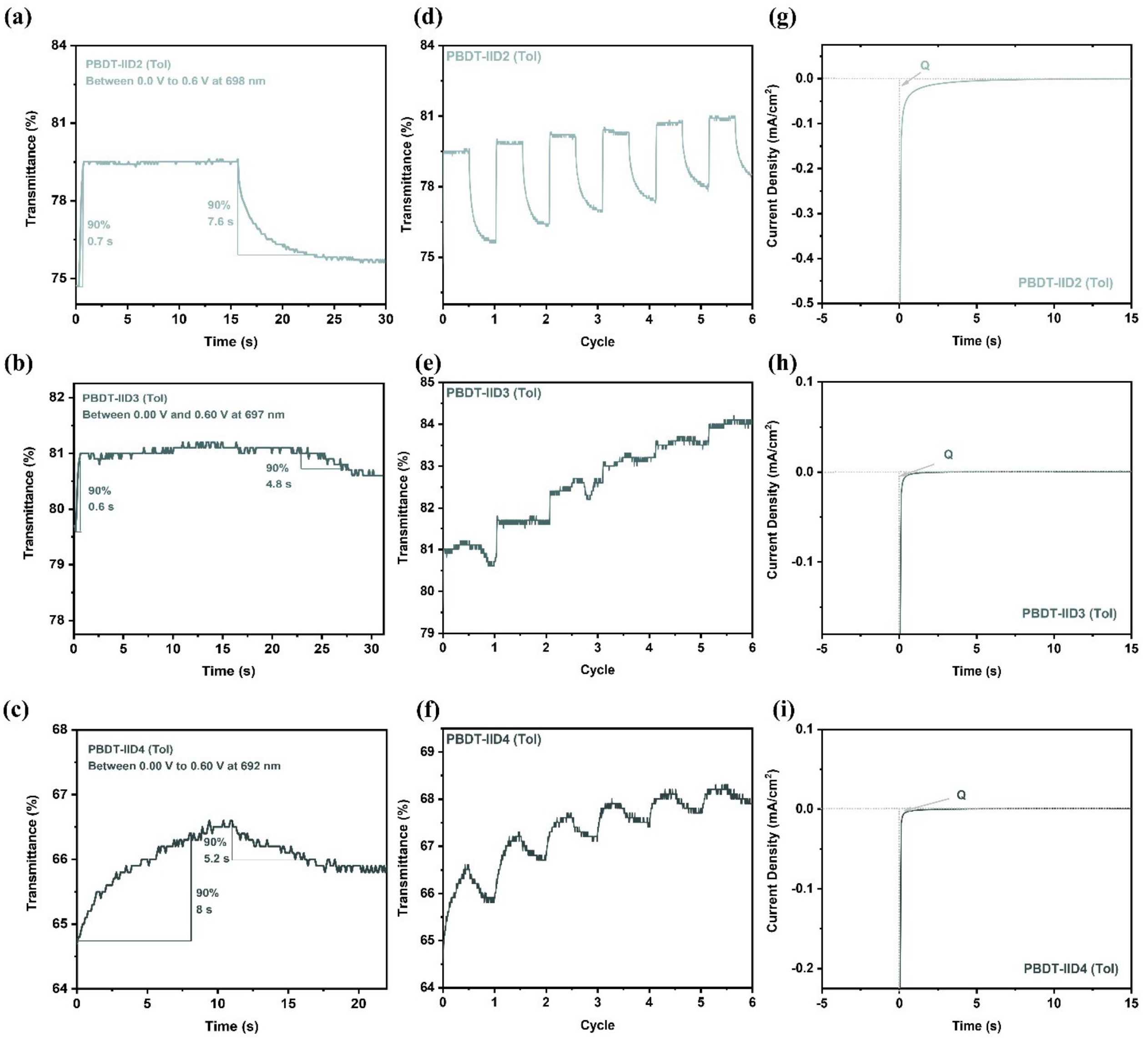

Figure S6. (a–c) Coloring/bleaching time and optical contrast of PBDT-IID2, PBDT-IID3 and PBDT-IID4; (d–f) Variation of optical contrast with cycle number for PBDT-IID2, PBDT-IID3 and PBDT-IID4; (g–i) Current versus time curves of PBDT-IID2, PBDT-IID3 and PBDT-IID4 when coloration occurs. (The processing solvent for polymer films is toluene.)

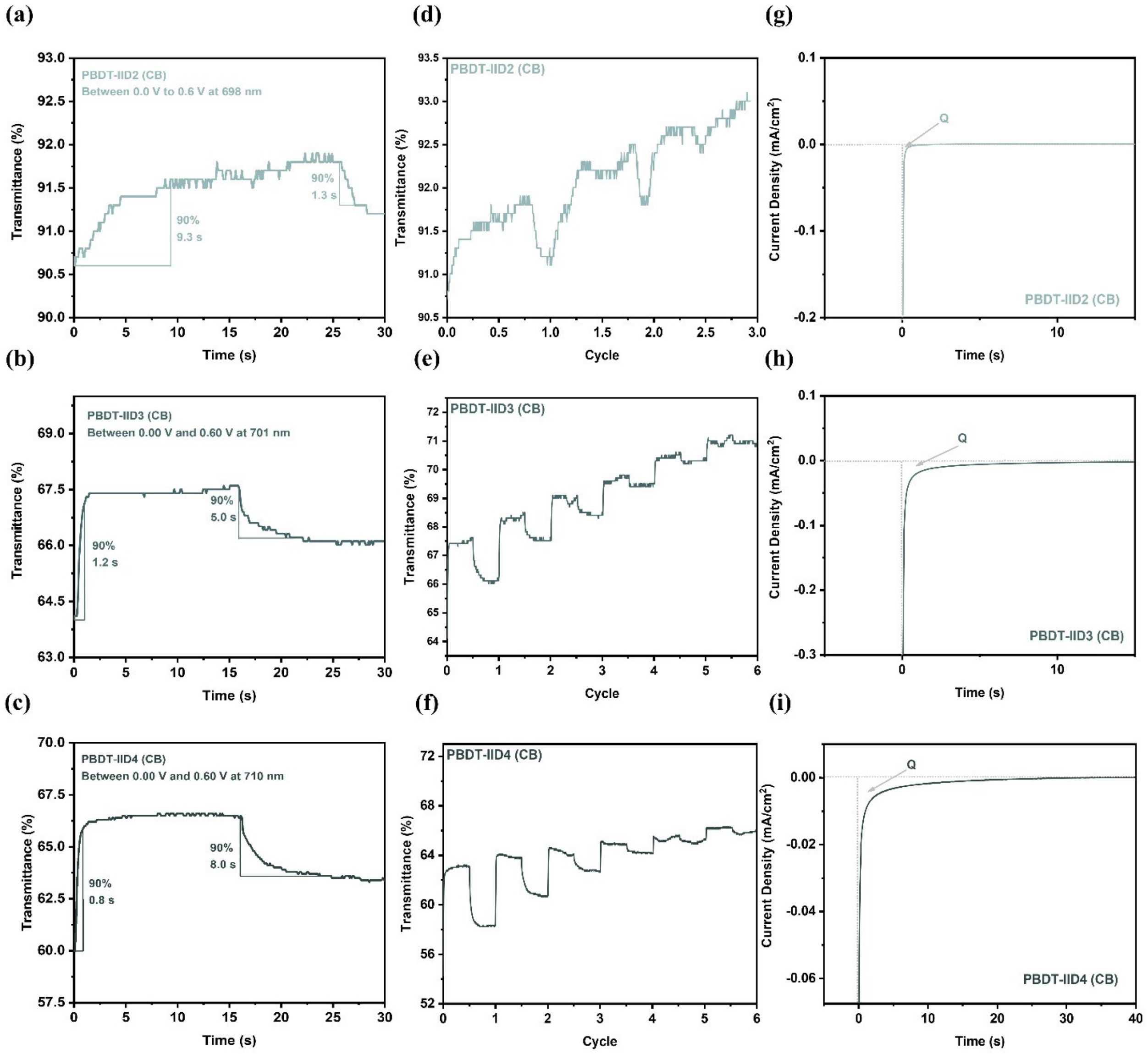

Figure S7. (a–c) Coloring/bleaching time and optical contrast of PBDT-IID2, PBDT-IID3 and PBDT-IID4; (d–f) Variation of optical contrast with cycle number for PBDT-IID2, PBDT-IID3 and PBDT-IID4; (g–i) Current versus time curves of PBDT-IID2, PBDT-IID3 and PBDT-IID4 when coloration occurs. (The processing solvent for polymer films is Chlorobenzene.)

Table S1. Solubility of PBDT-IIDs in non-halogen solvents.

Solvent	H_2O	MeOH	EtOH	Acetone	CPME
**PBDT-IID2**	--	--	--	--	--
**PBDT-IID3**	--	--	--	--	--
**PBDT-IID4**	--	--	--	--	+-
**Solvent**	**THF**	**2-MeTHF**	**Limonene**	**Toluene**	**DMF**
**PBDT-IID2**	+-	+-	--	++	--
**PBDT-IID3**	++	+-	--	++	--
**PBDT-IID4**	++	++	+-	++	+-

++: Soluble at room temperature.
+-: soluble on heating.
--: Insoluble on heating.
Qualitative solubility was determined with as 10 mg of polymer in 1 mL of solvent.

Table S2. Color coordinates of PBDT-IIDs films in its neutral and oxidized states using different processing solvents.

Polymer		Neutral state			Oxidation state		
		L	a*	b*	L	a*	b*
CB	PBDT-IID2	71.96	-9.05	-0.76	67.03	-3.30	7.56
	PBDT-IID3	75.09	-9.52	-2.40	68.12	-4.04	10.16
	PBDT-IID4	45.39	-4.94	-8.19	57.51	-0.58	8.25
Tol	PBDT-IID2	51.80	-6.96	3.72	69.41	-2.84	10.27
	PBDT-IID3	55.51	-7.56	4.18	64.53	-2.94	12.88
	PBDT-IID4	70.28	-12.00	-4.82	79.46	-3.04	-0.31
Me-THF	PBDT-IID2	37.58	-4.67	-8.91	50.08	9.05	24.25
	PBDT-IID3	72.35	-9.43	-3.60	78.42	-2.40	-2.40
	PBDT-IID4	62.94	-9.63	6.67	67.17	-3.16	11.14

Table S3. Detailed electrochromic data of PBDT-IIDs under different process solvents.

Solvents	Polymers	ΔT (%)	Response time (s) t_c/t_b
Tol	PBDT-IID2	5.1	0.7 s/7.6 s
	PBDT-IID3	1.4	0.6 s/4.8 s
	PBDT-IID4	1.75	8.0 s/5.2 s
CB	PBDT-IID2	1.15	9.3 s/1.3 s
	PBDT-IID3	3.6	1.2 s/5.0 s
	PBDT-IID4	6.3	0.8 s/8.0 s
